# Supplementary material for: Three-dimensional imaging through scattering media based on confocal diffuse tomography
Source: Nat Commun. 2020 Sep 9;11:4517. doi: 10.1038/s41467-020-18346-3 (PMC7481188; doi:10.1038/s41467-020-18346-3)
Supplement: Supplementary file 1 — Supplementary Information [file 41467_2020_18346_MOESM1_ESM.pdf]

Supplementary Information for

Three-dimensional imaging through scattering media based on confocal diffuse tomography

David B. Lindell, Gordon Wetzstein

Department of Electrical Engineering, Stanford University
350 Jane Stanford Way, Stanford, CA, 94305

This PDF file includes:

Supplementary Note 1: Calibration of the scattering layer
Supplementary Note 2: Image formation model
Supplementary Note 3: *f–k* migration
Supplementary Note 4: Closed-form inversion procedure
Supplementary Note 5: Iterative inversion procedure
Supplementary Note 6: Simulated results
Supplementary Note 7: Additional captured results
Supplementary Note 8: Resolution limits
Supplementary Figures 1 to 17
Supplementary Tables 1 to 2
Supplementary References

Other supplementary material for this manuscript includes the following:

Supplementary Movies 1 to 2
Supplementary Data 1

Supplementary Note 1: Calibration of the scattering layer

Measurement are acquired using a hardware prototype (shown in Supplementary Fig. 1) whose main components are an ultra-fast pulsed laser (NKT Katana 05HP) and a single-photon avalanche diode (Micro Photon Devices PDM Series Fast-Gated SPAD, $50 \times 50\,\mu$m active area) sharing an optical path through a polarizing beamsplitter (Thorlabs PBS251) and a 50 mm objective lens (Nikon Nikkor f/1.4). The pulsed laser source has a wavelength of 532 nm and is configured for a pulse repetition rate of 10 MHz with 400 mW average power. The system scans out an area on the surface of the scattering medium using a two-axis galvanometer (Thorlabs GVS012) whose scanning pattern is controlled by a National Instruments data acquisition device (NI-DAQ USB-6343). We use the gating capability of the SPAD to turn the detector on just before scattered photons arrive from the hidden object, and detected photons are timestamped using a time-correlated single-photon counter or TCSPC (PicoQuant PicoHarp 300). The combined timing resolution of the system is approximately 70 ps. The timestamps are post-processed into histograms before further processing with the reconstruction algorithm.

The reduced scattering (μ_s') and absorption (μ_a) coefficients of the scattering layer are calibrated by illuminating the scattering layer from one side using the pulsed laser and measuring the temporal response of the transmitted light at the other side with a single-pixel SPAD detector (Micro Photon Devices PDM series free-running SPAD). We capture 15 measurements with varying thicknesses of the scattering layer, from approximately 2.54 cm to 20.32 cm in increments of 1.27 cm. To model the measurements, we convolve the temporal response of the laser and SPAD with the solution of the time-dependent diffusion equation for a diffusing slab, described in the following section. We then optimize for the values of μ_s' and μ_a that minimize the mean squared error between the model and all measurements. During the optimization, the model and measurement values are normalized to a maximum value of 1. We also optimize an additional per-measurement time offset term which allows the model to account for any drift in the laser timing between capture of subsequent measurements. A non-linear iterative optimization procedure is implemented in PyTorch[1] using the AMSGrad optimizer[2] which iterates until the mean squared error between successive iterations falls below 10^{-6} or 5000 iterations are reached.

A main source of uncertainty in the calibrated measurement values is a model parameter called the extrapolation length. This parameter gives the location of an approximate boundary condition where the average diffusive intensity is equal to zero.[3] As we detail in the following section, the extrapolation length can be shown to depend on the refractive index of the medium,[4] which for the polyurethane foam used in our experiments is generally small due its low density.[5] While we find that a refractive index value of 1.12 produces the best fit to the data, we attempt to quantify the uncertainty in the calibrated values caused by a deviation in the refractive index and thus extrapolation length. To this end, we run the optimization for refractive index values across a range within $\pm 10\%$ of the nominal value (from 1.01 to 1.23 in increments of 0.01). Given that the outcome of the non-linear optimization could depend on the values used for

initialization, we also run each optimization for a range of initializations of μ_s' from 2.0 cm^{-1} to 3.0 cm^{-1} in increments of 0.1 cm^{-1}. The result is found to be relatively insensitive to the value of the absorption coefficient, which we initialize to zero.

We find that the minimum mean squared error is achieved with a refractive index of 1.12, a reduced scattering coefficient of 2.62 cm^{-1}, and an absorption coefficient of 5.29×10^{-3} cm^{-1}. These parameters provide a good fit to all recorded measurements as shown in Supplementary Fig. 2. A scatterplot of the mean-squared error and refractive index against the reduced scattering and absorption coefficients for all optimizations ($N = 253$) is shown in Supplementary Fig. 3. Here, we find that $\mu_s' = 2.62 \pm 0.43$ cm^{-1} and $\mu_a = 5.26 \times 10^{-3} \pm 5.5 \times 10^{-5}$ cm^{-1}, where the bounds indicate the range containing 95% of all optimized values.

Supplementary Note 2: Image formation model

Light transport for the problem of imaging through scattering medium can be decomposed into two components: the first describes propagation of light through a thick scattering layer, and the second models the free space propagation of light from the far side of the scattering layer to the hidden object and back.

Propagation through the scattering layer. To model light transport through the scattering medium, we solve the diffusion equation for the slab geometry of our setup. In this geometry, the physical interface between the scattering medium and the surrounding environment imposes boundary conditions that must be considered. A common approximation is to use an extrapolated boundary condition where the diffusive intensity is assumed to be zero at a flat surface located some extrapolation distance, z_e, away from either side of the slab. In other words, for a slab of thickness z_d, this condition states that the diffusive intensity is zero at $z = -z_\mathrm{e}$ and $z = z_\mathrm{d} + z_\mathrm{e}$ (see Supplementary Fig. 4). As we detail below, the value of z_e depends on the amount of internal reflection of diffusive intensity due to the refractive index mismatch at the medium-air interface.[6] To simplify the solution, we further assume that incident photons from a collimated beam of light are initially scattered isotropically at a distance $z_0 = 1/\mu'_\mathrm{s}$ into the scattering medium.[6–8]

The solution of the diffusion equation satisfies the extrapolated boundary condition by placing a positive and negative (dipole) source about $z = -z_\mathrm{e}$ such that the total diffusive intensity at the extrapolation distance is zero. However, a single dipole source does not satisfy the boundary condition at $z = z_\mathrm{d} + z_\mathrm{e}$. Instead, an infinite number of dipole sources is required, where the dipole of the near interface ($z = -z_\mathrm{e}$) is mirrored about the far interface, which is then mirrored about the near interface, and so on, as illustrated in Supplementary Fig. 4. The positions of these positive and negative sources are[3]

$$\begin{aligned} z_{+,i} &= 2i(z_\mathrm{d} + 2z_\mathrm{e}) + z_0 \\ z_{-,i} &= 2i(z_\mathrm{d} + 2z_\mathrm{e}) - 2z_\mathrm{e} - z_0 \, . \\ i &= 0, \pm 1, \pm 2, \ldots \end{aligned} \tag{1}$$

The resulting solution to the diffusion equation is[3,6]

$$\begin{aligned} \phi(t, \mathbf{r_0}, \mathbf{r_1}) = {}& \frac{1}{2(4\pi Dc)^{3/2}\, t^{5/2}} \exp\left(-\mu_\mathrm{a} ct - \frac{(r_{1,x} - r_{0,x})^2 + (r_{1,y} - r_{0,y})^2}{4Dct} \right) \\ & \cdot \sum_{i=-\infty}^{\infty} \left[(z_\mathrm{d} - z_{+,i}) \exp\left(-\frac{(z_\mathrm{d} - z_{+,i})^2}{4Dct} \right) - (z_\mathrm{d} - z_{-,i}) \exp\left(-\frac{(z_\mathrm{d} - z_{-,i})^2}{4Dct} \right) \right] , \end{aligned} \tag{2}$$

where ϕ is the power transmitted through the slab per unit area, $\mathbf{r_0} \in \Omega_0 = \{(r_{0,x}, r_{0,y}, r_{0,z}) \in \mathbb{R} \times \mathbb{R} \times \mathbb{R} \,|\, r_{0,z} = 0\}$ is the position illuminated by the laser and imaged by the detector, and

$\mathbf{r_1} \in \Omega_{z_\mathrm{d}} = \{(r_{1,x}, r_{1,y}, r_{1,z}) \in \mathbb{R} \times \mathbb{R} \times \mathbb{R} \,|\, r_{1,z} = z_\mathrm{d}\}$ is a spatial position on the far side of the scattering medium. We also have that c and t are the speed of light within the medium and time, respectively, and D is the diffusion coefficient, given by $D = (3(\mu_\mathrm{a} + \mu_\mathrm{s}'))^{-1}$. Generally, truncating the solution to 7 dipole pairs (i.e., $i = 0, \pm 1, \pm 2, \pm 3$) is sufficient to reduce the error to a negligible value.[3]

In this model, a challenging parameter to estimate is the extrapolation distance, z_e. The parameter depends on the reflection coefficient, R, of the scattering layer as[4]

$$z_\mathrm{e} = \frac{2l^*}{3} \frac{1+R}{1-R}, \tag{3}$$

where $l^* = 1/(\mu_\mathrm{a} + \mu_\mathrm{s}')$ is the transport mean free path. Zhu et al.[4] show that the value of the reflection coefficient is related to the refractive index of the scattering medium as follows. For an angle of incidence θ, the reflection coefficient averaged over polarization is given by

$$\bar{R}(\theta) = \frac{R_\perp(\theta) + R_\|(\theta)}{2}, \tag{4}$$

where $R_\perp(\theta)$ and $R_\|(\theta)$ are the Fresnel reflection coefficients. Then, let

$$C_1 = \int_0^{\pi/2} \bar{R}(\theta) \sin(\theta) \cos(\theta) d\theta \tag{5}$$

and

$$C_2 = \int_{-\pi/2}^{0} \bar{R}(\theta) \sin(\theta) \cos^2(\theta) d\theta. \tag{6}$$

Finally, an expression for R can be given as[4]

$$R = \frac{3C_2 + 2C_1}{3C_2 - 2C_1 + 2}. \tag{7}$$

Thus, for the refractive index of 1.12 and reduced scattering coefficient of 2.62 cm^{-1} that produce the best fit for our data, we find the reflection coefficient to have a value of 0.18 and the extrapolation distance to be $z_\mathrm{e} = 3.6$ mm.

Free space propagation. Using the solution to the diffusion equation allows us to model light transport through the scattering medium, but the complete measurement model must also describe the free space propagation of light from the surface of the scattering layer to a hidden object and back. This has been a subject of active research for the application of non-line-of-sight (NLOS) imaging,[9] and we can adopt a similar model to describe how light propagates from the far side of the scattering medium to the hidden object and back:[10,11]

$$I(t, \mathbf{r_1}, \mathbf{r_2}) = \int_\Psi f(\mathbf{x}, \mathbf{r_1}) f(\mathbf{x}, \mathbf{r_2}) \delta(ct - \|\mathbf{x} - \mathbf{r_1}\| - \|\mathbf{x} - \mathbf{r_2}\|)\, d\mathbf{x}. \tag{8}$$

Here, I is the photon flux at a point $\mathbf{r_2} \in \Omega_{z_\text{d}}$ resulting from light emitted at a location $\mathbf{r_1} \in \Omega_{z_\text{d}}$ and backscattered from the hidden object. The function f models the bidirectional scattering distribution function (BSDF) as well as albedo, visibility, and inverse-square falloff factors from a point $\mathbf{x}$ on the hidden object to a point on the boundary, Ω_{z_d} of the scattering medium. A delta function, δ relates distance and propagation time. The integration is performed over the hidden volume $\mathbf{x} \in \Psi = \{(x, y, z) \in \mathbb{R} \times \mathbb{R} \times \mathbb{R} \,|\, z \geq z_\text{d}\}$.

The time-resolved measurement at each sampled spatial location on the near-side of the scattering medium can be computed by first performing a temporal convolution of the diffusion impulse response ϕ from Supplementary Equation (2) with the free space propagation response given by I in Supplementary Equation (8). A second convolution with ϕ models diffusion back through the scattering medium to the point imaged by the detector. The complete measurement model can then be described as

$$\begin{aligned} \tau(t, \mathbf{r_0}) = &\int_{\Omega_{z_\text{d}}} \int_0^\infty \phi(t - t'', \mathbf{r_0}, \mathbf{r_2}) \\ &\cdot \left[\int_{\Omega_{z_\text{d}}} \int_0^\infty \phi(t'' - t', \mathbf{r_0}, \mathbf{r_1}) \cdot I(t', \mathbf{r_1}, \mathbf{r_2}) \, dt' d\mathbf{r_1} \right] dt'' d\mathbf{r_2}. \end{aligned} \tag{9}$$

Here, the innermost integral convolves light which diffuses through the scattering medium with the temporal response of the free space propagation to the hidden object and back. The result is integrated over all light-emitting positions on the far-side of the scattering medium, and then an additional convolution with Supplementary Equation (2) produces the photon flux on the observed side of the scattering medium. In practice, we simulate I using a custom implementation of a physically-based ray tracer[12] that relies on time-resolved bidirectional path tracing.

Efficient approximated image formation. Evaluating the forward model described by Supplementary Equation (9) is challenging because of the computation required to evaluate the innermost integral: we are required to evaluate all light paths from $\mathbf{r_1} \to \mathbf{x} \to \mathbf{r_2}$ in the expression for the free space photon flux I. However, a simplifying approximation can be made by observing that the concentrated light pulse transmitted through the scattering layer illuminates a spot on the far side whose full width at half-maximum is typically much smaller than the distance to the hidden object. This allows us to approximate light paths originating from $\mathbf{r_1}$ that return to a nearby location $\mathbf{r_2}$ with light paths that return back to the same location $\mathbf{r_1}$. In other words, we make the approximation $\mathbf{r_1} \approx \mathbf{r_2}$. See the following section for an extended analysis of this approximation.

For notational convenience, we give an alternate definition for the solution of the diffusion

equation (Supplemental Equation 2), where the diffuser thickness z_d is a constant.

$$\begin{aligned}\phi_{z_\mathrm{d}}(t, \bar{\mathbf{r}}_\mathbf{0}, \bar{\mathbf{r}}_\mathbf{1}) = &\frac{\exp\left(-\mu_\mathrm{a} ct - \frac{\|\bar{\mathbf{r}}_\mathbf{1} - \bar{\mathbf{r}}_\mathbf{0}\|)^2}{4Dct}\right)}{2(4\pi Dc)^{3/2} t^{5/2}} \\ &\cdot \sum_{i=-\infty}^{\infty} \left[z_{1,i} \exp\left(-\frac{z_{1,i}^2}{4Dct}\right) - z_{2,i} \exp\left(-\frac{z_{2,i}^2}{4Dct}\right)\right],\end{aligned} \tag{10}$$

where $\bar{\mathbf{r}}_{\mathbf{0/1}} \in \Omega = \{(r_{0/1,x}, r_{0/1,y}) \in \mathbb{R} \times \mathbb{R}\}$, or we simply factor z_d into the diffusion equation instead of incorporating it into $\mathbf{r_{0/1}}$. The resulting simplified expression for Supplementary Equation (9) is

$$\begin{aligned}\hat{\tau}(t, \mathbf{r_0} = (\bar{\mathbf{r}}_\mathbf{0}, z_\mathrm{d})) = &\int_\Omega \int_0^\infty \phi_{z_\mathrm{d}}(t - t'', \bar{\mathbf{r}}_\mathbf{0} - \bar{\mathbf{r}}') \\ &\cdot \left[\int_\Omega \int_0^\infty \phi_{z_\mathrm{d}}(t'' - t', \bar{\mathbf{r}}' - \bar{\mathbf{r}}_\mathbf{1}) \cdot I\left(t', \underbrace{(\bar{\mathbf{r}}_\mathbf{1}, z_\mathrm{d})}_{\in \Omega_{z_\mathrm{d}}}, \underbrace{(\bar{\mathbf{r}}_\mathbf{1}, z_\mathrm{d})}_{\in \Omega_{z_\mathrm{d}}}\right) dt' d\bar{\mathbf{r}}_\mathbf{1}\right] dt'' d\bar{\mathbf{r}}'.\end{aligned} \tag{11}$$

The mixing of light paths in the local area of $\mathbf{r_1}$ is modeled by spatial convolution with the diffusion kernel, and propagation back through the scattering medium is modeled with another convolution with the diffusion kernel. This formulation can be expressed more simply as

$$\begin{aligned}\hat{\tau}(t, \mathbf{r_0}) &= \phi(t, \mathbf{r_0}, \mathbf{r_1}) * \phi(t, \mathbf{r_0}, \mathbf{r_1}) * I(t, \mathbf{r_1}, \mathbf{r_1}) \\ &= \bar{\phi} * I,\end{aligned} \tag{12}$$

where '$*$' denotes convolution. Here, the scattering kernel $\bar{\phi} = \phi(t, \mathbf{r_0}, \mathbf{r_1}) * \phi(t, \mathbf{r_0}, \mathbf{r_1})$ is calculated based on the reduced scattering and absorption coefficients and the thickness of the scattering layer, and $I(t, \mathbf{r_1}, \mathbf{r_1})$ is equivalent to a confocal measurement in NLOS imaging.[13] While "confocalizing" the measurements, so that $I(t, \mathbf{r_1}, \mathbf{r_2})$ is replaced by $I(t, \mathbf{r_1}, \mathbf{r_1})$, simplifies the forward model, another key benefit is that such confocal measurements can be inverted efficiently to recover a hidden surface.[13,14]

Approximated image formation error. In the forward image formation model described by Supplementary Equation (12), the free space light transport $I(t, \mathbf{r_1}, \mathbf{r_2})$ is approximated by $I(t, \mathbf{r_1}, \mathbf{r_1})$. The error in this approximation results from the difference in the round-trip path length between $\mathbf{r_1} \to \mathbf{x} \to \mathbf{r_2}$ and $\mathbf{r_1} \to \mathbf{x} \to \mathbf{r_1}$, given as

$$\begin{aligned}&\underbrace{\|\mathbf{r_1} - \mathbf{x}\| + \|\mathbf{r_2} - \mathbf{x}\|}_{\mathbf{r_1} \to \mathbf{x} \to \mathbf{r_2}} - \underbrace{2\|\mathbf{r_1} - \mathbf{x}}_{\mathbf{r_1} \to \mathbf{x} \to \mathbf{r_1}}\| \\ &= \|\mathbf{r_2} - \mathbf{x}\| - \|\mathbf{r_1} - \mathbf{x}\| \\ &= d_2 - d_1\end{aligned} \tag{13}$$

For values of $\mathbf{r_1}$ and $\mathbf{r_2}$ that make a small angle with a point $\mathbf{x}$ at a standoff distance, H (see Supplementary Fig. 5b), we can express the error using a paraxial approximation. Let L be the lateral distance between $\mathbf{r_1}$ and $\mathbf{r_2}$ and let δ be the difference $d_2 - d_1$; then for $L \ll H$ we have that

$$\begin{aligned} &H + \delta = \sqrt{L^2 + H^2} \\ &\Rightarrow H + \delta \approx H + \frac{L^2}{2H} \\ &\Rightarrow \delta \approx \frac{L^2}{2H}, \end{aligned} \tag{14}$$

where the second step uses the generalized binomial theorem. The lateral distance L corresponds to the spot size illuminated on the far side of the scattering medium, which scales as the thickness of the scattering layer, z_d.[15] Thus the approximation error decreases with increasing standoff distance or decreasing spot size or scattering layer thickness. In general, the approximation has negligible effect on the reconstruction when the error is less than the resolution of the imaging system, or $c\Delta t_\text{system} > \frac{L^2}{2H}$, where Δt_system is the system temporal resolution.

For our experimental setup, the full width at half maximum (FWHM) of the illuminated spot size at the far side of the scattering layer is approximately 2.2 cm, which is roughly the same as the 2.5 cm thickness of the scattering layer. We show the measured FWHM beamwidth in Supplementary Fig. 5. At very large incidence angles, such as might occur with large scan areas and a small standoff distance, the maximum error is equal to the lateral distance L. We show the exact error and the regime where the paraxial approximation applies for a range of hidden object positions in Supplementary Fig. 5. Given the 2 cm resolution of our hardware prototype system, the approximation should not significantly degrade the reconstruction quality even outside the paraxial regime.

We also illustrate the difference between measurements simulated for a scene containing a letter "S" using Supplementary Equation (9) and Supplementary Equation (11). The measurements are normalized to a maximum value of one and we illustrate the differences in a maximum projection visualization. We calculate the mean absolute error between the normalized the measurements from the full and approximate image-formation models, yielding a value of 0.007 (arbitrary unit) with the largest error occurring near the volume boundaries (see Supplementary Fig. 6).

Supplementary Note 3: *f–k* migration

Frequency–wavenumber (*f–k*) migration is a technique used for image reconstruction in a number of sensing modalities, including seismic imaging[16] and synthetic aperture radar.[17] This inversion technique can similarly be applied to confocal measurements of light scattering from a point on a surface to a hidden object and back to the same point, as in NLOS imaging.[14] In our application, we apply *f–k* migration to invert the scattering of light from the far side of the scattering medium to the hidden object and back. We briefly overview the technique here and refer the interested reader to more detailed treatments provided by Lindell et al. in the context of NLOS imaging[14] or Margrave and Lamoureux for seismic imaging.[18]

In our approximated image formation model, the confocal measurements capture light that travels along specific paths: from a point on the far side of the scattering medium to the hidden object and back to the same point. In other words, the captured photons undergo two-way propagation to the hidden object and back along the same path. Rather than modeling this two-way propagation of light, we can use an equivalent one-way propagation model where measurements arise from a pulsed wavefront of light that is emitted simultaneously from each point on the surface of the hidden object and propagates to the far side of the scattering medium at half the speed of light. This is known as the "exploding reflector model"[18] in seismic imaging. With this model, reconstructing the hidden object geometry consists of migrating the measured wavefront back to its origin, where it outlines the surface of the hidden object.

To migrate the measurements, we treat the envelope of light "emitted" by the hidden object as a wave[19,20] and model its propagation with the wave equation

$$\left(\nabla^2 - \frac{1}{c^2}\frac{\partial^2}{\partial t^2}\right)\xi(x, y, z, t) = 0, \tag{15}$$

where ∇^2 is the spatial Laplacian, c is the speed of light, and ξ is a scalar wavefield defined over three spatial dimensions and time. We assume that the wavefront of light is emitted by the hidden object at a time $t = 0$ and that measurements are captured on a plane at $z = 0$. Then, the wavefield ξ can be written as an integral over plane waves at differential spatiotemporal frequencies:

$$\xi(x, y, z, t) = \iiint \Xi(k_x, k_y, k_z) e^{2\pi i(k_x x + k_y y + k_z z - \nu t)} dk_x dk_y dk_z. \tag{16}$$

Here, the wavenumber is given by $\mathbf{k} = 2\pi \cdot (k_x, k_y, k_z)$, temporal frequency is represented by ν, and Ξ is a complex-valued number which represents the amplitude and phase of each plane wave at $t = 0$. Note that ξ and Ξ are related through a Fourier transform when $t = 0$.

We can derive a similar relationship that relates ξ to a transformed version of Ξ through a Fourier transform at $z = 0$. Then, the two relations are used to efficiently migrate the wavefield from measurements captured at $z = 0$ to the surface of the hidden object at $t = 0$. Using the

dispersion relation, we can relate temporal frequency ν to the wavenumber as

$$\nu = c\sqrt{k_x^2 + k_y^2 + k_z^2}. \tag{17}$$

With a substitution of variables, we can rewrite Supplementary Equation (16) as

$$\xi(x, y, z, t) = \iiint \bar{\Xi}(k_x, k_y, \nu) e^{2\pi i(k_x x + k_y y + k_z z - \nu t)} dk_x dk_y d\nu, \tag{18}$$

and the following expression relates Ξ and $\bar{\Xi}$ using the dispersion relation and its Jacobian.

$$\Xi(k_x, k_y, k_z) = \frac{c|k_z|}{\sqrt{k_x^2 + k_y^2 + k_z^2}} \bar{\Xi}\left(k_x, k_y, c\sqrt{k_x^2 + k_y^2 + k_z^2}\right). \tag{19}$$

Note that in Supplementary Equation (18), ξ and $\bar{\Xi}$ are related through a Fourier transform at $z = 0$.

With these equations, migration of the wavefield from the measurements at $z = 0$ to the surface of the hidden object at $t = 0$ can be accomplished in three steps by first, taking the Fourier transform of the measurements, second, performing an interpolation operation described by Supplementary Equation (19), and third, performing an inverse Fourier transform. With this straightforward reconstruction procedure, *f–k* migration is computationally efficient and relatively uncomplicated to implement in practice (see, e.g., pseudocode in Supplementary Note 4).

Supplementary Note 4: Closed-form inversion procedure

Discretizing the image formation. In practice the image formation model is implemented numerically with discrete operations. The spatiotemporal measurements of photon flux are discretized as

$$(\hat{\boldsymbol{\tau}})_{i,j,k} = \int_{y_{k-1}}^{y_k} \int_{x_{j-1}}^{x_j} \int_{t_{i-1}}^{t_i} \hat{\tau}\left(t', \mathbf{r_0}' = (x', y', 0)\right) \, dt' dx' dy', \tag{20}$$

where $1 \leq i \leq n_t$, $1 \leq j \leq n_x$, and $1 \leq k \leq n_y$. The time values t_i evenly divide a range $[a, b] \in (0, \infty)$, the spatial values similarly divide the spatial domain of the sampled region $\mathbf{\Omega_0}$, and $\hat{\boldsymbol{\tau}}$ is a vectorized measurement.

The continuous convolution operator $\bar{\phi}$ and the continuous free space propagation operator I are also implemented with discrete operations in practice. We denote the discrete diffusion operator as the convolution matrix (or its equivalent matrix-free operation) $\bar{\mathbf{\Phi}}$. We let $\mathbf{A}$ be the matrix that describes free space propagation to the hidden object and back. The full discretized image formation model is then given as

$$\hat{\boldsymbol{\tau}} = \bar{\mathbf{\Phi}} \mathbf{A} \boldsymbol{\rho}, \tag{21}$$

where $\boldsymbol{\rho}$ is the sought-after albedo of the hidden object.

Closed-form solution. Given an ideal photon counting detector that sees an average photon arrival rate of $\boldsymbol{\tau}$, the probability of detecting a certain number of photon events in a discrete spatiotemporal interval is Poisson distributed and given as[21]

$$(\tilde{\boldsymbol{\tau}})_{i,j,k} \sim \mathcal{P}\left((\boldsymbol{\tau})_{i,j,k}\right). \tag{22}$$

For measurements with high photon counts, we can approximate the Poisson measurement noise as additive Gaussian noise such that

$$\begin{aligned} (\tilde{\boldsymbol{\tau}})_{i,j,k} &\approx (\boldsymbol{\tau})_{i,j,k} + \eta \\ \eta &\sim \mathcal{N}((\boldsymbol{\tau})_{i,j,k}, (\boldsymbol{\tau})_{i,j,k}). \end{aligned} \tag{23}$$

In this case, a closed-form solution exists using the Wiener deconvolution filter and a confocal inverse filter $\mathbf{A}^{-1}$ used in NLOS imaging (e.g., the Light Cone Transform[13,22] or *f-k* migration[14]):

$$\hat{\boldsymbol{\rho}} = \mathbf{A}^{-1} \mathbf{F}^{-1} \left[\frac{\hat{\bar{\mathbf{\Phi}}}^*}{|\hat{\bar{\mathbf{\Phi}}}|^2 + \frac{1}{\alpha}} \right] \mathbf{F} \tilde{\boldsymbol{\tau}}, \tag{24}$$

where α is a parameter that varies depending on the signal-to-noise ratio at each frequency, $\hat{\bar{\mathbf{\Phi}}}$ is the diagonal matrix with the Fourier coefficients of the 3D convolution, and $\mathbf{F}$ denotes the discrete Fourier transform matrix.

We also provide code and data in Supplementary Data 1 which implements the closed-form solution and reproduces results from the main text. Code and data are also available online at https://github.com/computational-imaging/confocal-diffuse-tomography, and we provide a pseudocode implementation below.

Algorithm Confocal diffuse tomography

---

procedure CDT($\tilde{\boldsymbol{\tau}}(x, y, t)$)

    // Initialize SNR parameter

    $\alpha \leftarrow \alpha_0$

    // Pad measurements

    $\tilde{\boldsymbol{\tau}}(x, y, t) = pad\_volume(\tilde{\boldsymbol{\tau}}(x, y, t))$

    // Fast Fourier Transform

    $\boldsymbol{T}(k_x, k_y, \nu) = \mathcal{F}_{\{x,y,t\}} \{\tilde{\boldsymbol{\tau}}(x, y, t)\}$

    // Perform deconvolution with diffusion kernel

    $\boldsymbol{\xi}(x, y, t) = \mathcal{F}^{-1}_{\{x,y,t\}} \left\{ \left[\frac{\hat{\bar{\boldsymbol{\Phi}}}^*}{|\hat{\bar{\boldsymbol{\Phi}}}|^2 + \frac{1}{\alpha}} \right] \boldsymbol{T}(k_x, k_y, \nu) \right\}$

    // Perform *f–k* migration

    $\hat{\boldsymbol{\rho}}(x, y, z) =$ *f–k* MIGRATION($\boldsymbol{\xi}(x, y, t)$)

    // Unpad reconstruction

    $\hat{\boldsymbol{\rho}}(x, y, z) = unpad\_volume(\hat{\boldsymbol{\rho}}(x, y, z))$

    return $\hat{\boldsymbol{\rho}}(x, y, z)$

end procedure

function *f–k* MIGRATION($\boldsymbol{\xi}(x, y, t)$)

    // Pre-process data

    $\boldsymbol{\xi}(x, y, t) = t \cdot \sqrt{\boldsymbol{\xi}(x, y, t)}$

    // Fast Fourier transform

    $\bar{\Xi}(k_x, k_y, \nu) = \mathcal{F}_{\{x,y,t\}} \{\boldsymbol{\xi}(x, y, t)\}$

    // Interpolation

    $\Xi(k_x, k_y, k_z) = \frac{v|k_z|}{\sqrt{k_x^2 + k_y^2 + k_z^2}} \cdot resample\left(\bar{\Xi}(k_x, k_y, \nu)\right)$

    // Inverse Fast Fourier transform

    $\boldsymbol{\xi}(x, y, z) = \mathcal{F}^{-1}_{\{x,y,z\}} \{\Xi(k_x, k_y, k_z)\}$

    // Post-process data

    return $|\boldsymbol{\xi}(x, y, z)|^2$

end function

Supplementary Note 5: Iterative inversion procedure

Improved performance is achieved by accurately modeling the Poisson noise and the boundary conditions imposed by integrating the measurements over a finite volume. In this case, we use a maximum a posteriori estimator which incorporates the structure of the approximate forward image formation and accounts for the Poisson noise inherent in the measurements. To better handle the boundary conditions, we slightly amend the definition of the measurements $\tilde{\boldsymbol{\tau}}$ to include a cropping term which truncates the measurements to the physical area observed by the detector, and we expand the size of the reconstructed hidden volume to account for the large support of the scattering kernel. The revised formulation is

$$\hat{\boldsymbol{\tau}} = \mathbf{M}\bar{\boldsymbol{\Phi}}\mathbf{A}\boldsymbol{\rho}. \tag{25}$$

The matrix $\mathbf{M} \in \mathbb{Z}_2^{m\times n}$, $\mathbb{Z}_2 = \{0, 1\}$, is a diagonal cropping matrix with $m < n$ that crops the measurements to the appropriate area.

We minimize the negative log-likelihood of the image formation, given as

$$\begin{aligned} &\underset{\mathbf{x}}{\operatorname{argmin}} \quad -\log p(\tilde{\boldsymbol{\tau}}|\mathbf{M}\bar{\boldsymbol{\Phi}}\boldsymbol{\rho}_{\boldsymbol{A}}) + \lambda\Gamma(\mathbf{H}\boldsymbol{\rho}_{\boldsymbol{A}}) \\ &\text{s.t.} \quad \boldsymbol{\rho}_{\boldsymbol{A}} \geq 0 \end{aligned} \tag{26}$$

where we absorb the free space propagation operator into $\boldsymbol{\rho}_{\boldsymbol{A}} = \mathbf{A}\boldsymbol{\rho}$, Γ is a prior on $\boldsymbol{\rho}_{\boldsymbol{A}}$, $\mathbf{H}$ is a linear operator used in the prior function, and λ controls the strength of the prior. In order to reduce the computation required for the iterative inversion, we solve for the measurement estimate $\boldsymbol{\rho}_{\boldsymbol{A}}$ and then obtain a final estimate for the hidden albedo with a single application of the inverse free space propagation operator $\mathbf{A}^{-1}$.

We replace this objective with an equivalent, unconstrained cost function

$$\underset{\mathbf{x}}{\operatorname{argmin}} \quad -\log p(\tilde{\boldsymbol{\tau}}|\mathbf{M}\bar{\boldsymbol{\Phi}}\boldsymbol{\rho}_{\boldsymbol{A}}) + \lambda\Gamma(\mathbf{H}\boldsymbol{\rho}_{\boldsymbol{A}}) + \mathcal{I}_{\mathbb{R}_+}(\boldsymbol{\rho}_{\boldsymbol{A}}) \tag{27}$$

where

$$\mathcal{I}_{\mathbb{R}_+}(x) = \begin{cases} 0, & x \in \mathbb{R}_+ \\ +\infty, & x \notin \mathbb{R}_+ \end{cases} \tag{28}$$

is an indicator function and $\mathbb{R}_+$ denotes the convex set of non-negative, real numbers.

This problem is solved using the alternating direction method of multipliers (ADMM)[23] to split this problem into sub-problems and enforce consensus in the constraints. The problem is reformulated as

$$\begin{aligned} &\underset{\mathbf{x}}{\operatorname{argmin}} \quad -\log p(\tilde{\boldsymbol{\tau}}|\mathbf{M}\mathbf{z_1}) + \lambda\Gamma(\mathbf{z_2}) + \mathcal{I}_{\mathbb{R}_+}(\mathbf{z_3}) \\ &\text{s.t.} \quad \underbrace{\begin{bmatrix} \bar{\boldsymbol{\Phi}} \\ \mathbf{H} \\ \mathbf{I} \end{bmatrix}}_{\mathbf{K}} \boldsymbol{\rho}_{\boldsymbol{A}} = \underbrace{\begin{bmatrix} \mathbf{z_1} \\ \mathbf{z_2} \\ \mathbf{z_3} \end{bmatrix}}_{\mathbf{z}}, \end{aligned} \tag{29}$$

where $\mathbf{I}$ is the identity matrix.

We minimize the scaled form[23] of the augmented Lagrangian for this expression, which is

$$L_\rho(\boldsymbol{\rho_A}, \mathbf{z}, \mathbf{u}) = \underbrace{-\log p(\tilde{\boldsymbol{\tau}}|\mathrm{M}\mathbf{z_1})}_{g_1(\mathbf{z_1})} + \underbrace{\lambda\Gamma(\mathbf{z_2})}_{g_2(\mathbf{z_2})} + \underbrace{\mathcal{I}_{\mathbb{R}_+}(\mathbf{z_3})}_{g_3(\mathbf{z_3})} + \frac{\rho}{2}\|\mathbf{K}\boldsymbol{\rho_A} - \mathbf{z} + \mathbf{u}\|_2^2 - \frac{\rho}{2}\|\mathbf{u}\|_2^2. \quad (30)$$

An iterative solver is used to minimize this form and consists of the following repeated update steps.

$\boldsymbol{\rho_A} \leftarrow \boldsymbol{\rho_0},\ \mathbf{u} \leftarrow \mathbf{0},\ \mathbf{z} \leftarrow \mathbf{0}$

for $k = 1$ **to** max_iter

$$\boldsymbol{\rho_A} \leftarrow \mathrm{prox}_{\|\cdot\|_2}(\mathbf{v}) = \underset{\boldsymbol{\rho}_\mathrm{A}}{\mathrm{argmin}}\ \mathrm{L}_\rho(\boldsymbol{\rho}_\mathrm{A}, \mathbf{z}, \mathbf{u}) = \underset{\boldsymbol{\rho}_\mathrm{A}}{\mathrm{argmin}}\ \frac{1}{2}\|\mathbf{K}\boldsymbol{\rho}_\mathrm{A} - \mathbf{v}\|_2^2, \quad \mathbf{v} = \mathbf{z} - \mathbf{u} \quad (31)$$

$$\mathbf{z_1} \leftarrow \mathrm{prox}_{\mathcal{P},\rho}(\mathbf{v}) = \underset{\mathbf{z_1}}{\mathrm{argmin}}\ \mathrm{L}_\rho(\boldsymbol{\rho}_\mathrm{A}, \mathbf{z}, \mathbf{u}) = \underset{\mathbf{z_1}}{\mathrm{argmin}}\ \mathrm{g}_1(\mathbf{z_1}) + \frac{\rho}{2}\|\mathbf{v} - \mathbf{z_1}\|_2^2, \quad \mathbf{v} = \bar{\boldsymbol{\Phi}}\boldsymbol{\rho_A} + \mathbf{u_1} \quad (32)$$

$$\mathbf{z_2} \leftarrow \mathrm{prox}_{\Gamma,\rho}(\mathbf{v}) = \underset{\mathbf{z_2}}{\mathrm{argmin}}\ \mathrm{L}_\rho(\boldsymbol{\rho}_\mathrm{A}, \mathbf{z}, \mathbf{u}) = \underset{\mathbf{z_2}}{\mathrm{argmin}}\ \mathrm{g}_2(\mathbf{z_2}) + \frac{\rho}{2}\|\mathbf{v} - \mathbf{z_2}\|_2^2, \quad \mathbf{v} = \mathbf{H}\boldsymbol{\rho_A} + \mathbf{u_2} \quad (33)$$

$$\mathbf{z_3} \leftarrow \mathrm{prox}_{\mathcal{I},\rho}(\mathbf{v}) = \underset{\mathbf{z_3}}{\mathrm{argmin}}\ \mathrm{L}_\rho(\boldsymbol{\rho}_\mathrm{A}, \mathbf{z}, \mathbf{u}) = \underset{\mathbf{z_3}}{\mathrm{argmin}}\ \mathrm{g}_3(\mathbf{z_3}) + \frac{\rho}{2}\|\mathbf{v} - \mathbf{z_3}\|_2^2, \quad \mathbf{v} = \boldsymbol{\rho_A} + \mathbf{u_3} \quad (34)$$

$$\underbrace{\begin{bmatrix}\mathbf{u_1}\\ \mathbf{u_2}\\ \mathbf{u_3}\end{bmatrix}}_{\mathbf{u}} \leftarrow \mathbf{u} + \mathbf{K}\boldsymbol{\rho_A} - \mathbf{z} \quad (35)$$

end for

Proximal operator for the quadratic term. The proximal operator for the quadratic subproblem (Supplementary Equation (32)) has a closed-form solution given by the normal equations. The solution is

$$\mathrm{prox}_{\|\cdot\|_2}(\mathbf{v}) = \underset{\rho}{\mathrm{argmin}}\ \frac{1}{2}\|\mathbf{K}\boldsymbol{\rho_A} - \mathbf{v}\|_2^2 \quad (36)$$

$$= \left(\bar{\boldsymbol{\Phi}}^T\bar{\boldsymbol{\Phi}} + \mathbf{H}^T\mathbf{H} + \mathbf{I}^T\mathbf{I}\right)^{-1}\left(\bar{\boldsymbol{\Phi}}^T(\mathbf{z_1} - \mathbf{u_1}) + \mathbf{H}^T(\mathbf{z_2} - \mathbf{u_2}) + \mathbf{I}^T(\mathbf{z_3} - \mathbf{u_3})\right). \quad (37)$$

Since each submatrix in $\mathbf{K}$ is circulant, the solution can be computed efficiently using element-wise multiplications in the Fourier domain.

Proximal operator for the Poisson term. The update for the Poisson term (Supplementary Equation (33)) is given as

$$\underset{\mathbf{z_1}}{\text{argmin}} \quad -\log p(\tilde{\boldsymbol{\tau}}|\mathbf{M}\mathbf{z_1}) + \frac{\rho}{2}\|\mathbf{z_1} - \mathbf{v}\|_2^2.$$

Note that with the diagonal cropping matrix $\mathbf{M}$, values of $\mathbf{z_1}$ are simply copied or omitted, and so the objective function reduces to two cases. With $\mathbf{M} \in \mathbb{Z}_2^{m\times n}$ and $\mathbf{z_1} \in \mathbb{R}^n$, $\mathbf{M}$ copies $(\mathbf{z_1})_i$ for $1 \leq i \leq m$ and omits $(\mathbf{z_1})_i$ for $m+1 \leq i \leq n$. Since the probabilities of each $(z_i)_i$ are independent, we can use the elementwise negative log likelihood to write the objective as

$$\begin{cases} \underset{\mathbf{z_1}}{\text{argmin}} \;\; -(\log(\mathbf{z_1})_i)\,(\tilde{\boldsymbol{\tau}})_i + (\mathbf{z_1})_i - \log\dfrac{1}{(\tilde{\boldsymbol{\tau}})_i!} + \dfrac{\rho}{2}\left((\mathbf{z_1})_i - (\mathbf{v})_i\right)^2, & 1 \leq i \leq m \\ \underset{\mathbf{z_1}}{\text{argmin}} \;\; \dfrac{\rho}{2}\left((\mathbf{z_1})_i - (\mathbf{v})_i\right)^2, & m+1 \leq i \leq n \end{cases}$$

For the second case, we trivially have that

$$\text{prox}_{\mathcal{P},\rho}(\mathbf{v}) = \underset{\mathbf{z_1}}{\text{argmin}} \; \frac{\rho}{2}\left((\mathbf{z_1})_{\mathrm{i}} - (\mathbf{v})_{\mathrm{i}}\right)^2 = (\mathbf{v})_{\mathrm{i}}, \quad \mathrm{m}+1 \leq \mathrm{i} \leq \mathrm{n} \tag{38}$$

For the first case, we minimize the objective function by setting the derivative to zero and solving for the value of $\mathbf{z_1}$.

$$\frac{d}{d(\mathbf{z_1})_i}\left(-(\log(\mathbf{z_1})_i)\,(\tilde{\boldsymbol{\tau}})_i + (\mathbf{z_1})_i - \log\frac{1}{(\tilde{\boldsymbol{\tau}})_i!} + \frac{\rho}{2}\left((\mathbf{z_1})_i - (\mathbf{v})_i\right)^2\right) \tag{39}$$

$$= -\frac{(\tilde{\boldsymbol{\tau}})_i}{(\mathbf{z_1})_i} + 1 + \rho((\mathbf{z_1})_i - (\mathbf{v})_i) = 0 \tag{40}$$

$$\Rightarrow +(\mathbf{z_1})_i^2 + \left(\frac{1}{\rho} - (\mathbf{v})_i\right)(\mathbf{z_1})_i + \frac{(\tilde{\boldsymbol{\tau}})_i}{\rho} = 0 \tag{41}$$

This result is a quadratic expression with two solutions; however only the positive solution is physically realizable and is given as

$$\text{prox}_{\mathcal{P},\rho}(\mathbf{v}) = -\frac{1-\rho(\mathbf{v})_{\mathrm{i}}}{2\rho} + \sqrt{\left(\frac{1-\rho(\mathbf{v})_{\mathrm{i}}}{2\rho}\right) + \frac{(\tilde{\boldsymbol{\tau}})_{\mathrm{i}}}{\rho}}, \quad 1 \leq \mathrm{i} \leq \mathrm{m}. \tag{42}$$

Proximal operator for the prior term. For the prior term (Supplementary Equation (34)), we use the Frobenius norm of the Hessian which encourages piecewise smoothness in the estimated measurements. The Hessian operator is defined such that

$$(\mathcal{H}\boldsymbol{\rho_A})_j = \begin{bmatrix} (\mathbf{D_{xx}}\boldsymbol{\rho_A})_j & (\mathbf{D_{xy}}\boldsymbol{\rho_A})_j & (\mathbf{D_{xz}}\boldsymbol{\rho_A})_j \\ (\mathbf{D_{yx}}\boldsymbol{\rho_A})_j & (\mathbf{D_{yy}}\boldsymbol{\rho_A})_j & (\mathbf{D_{yz}}\boldsymbol{\rho_A})_j \\ (\mathbf{D_{zx}}\boldsymbol{\rho_A})_j & (\mathbf{D_{zy}}\boldsymbol{\rho_A})_j & (\mathbf{D_{zz}}\boldsymbol{\rho_A})_j \end{bmatrix} \tag{43}$$

where $\mathbf{D_{ij}} = \mathbf{D_i D_j}$ for $i, j \in \{x, y, z\}$ are linear finite difference operators. For convenience, we use the linear operator $\mathbf{H}$ given as

$$(\mathbf{H}\boldsymbol{\rho_A})_j = \begin{bmatrix} (\mathbf{D_{xx}}\boldsymbol{\rho_A})_j & (\mathbf{D_{yy}}\boldsymbol{\rho_A})_j & (\mathbf{D_{zz}}\boldsymbol{\rho_A})_j & (\mathbf{D_{xy}}\boldsymbol{\rho_A})_j & (\mathbf{D_{xz}}\boldsymbol{\rho_A})_j & (\mathbf{D_{yz}}\boldsymbol{\rho_A})_j \end{bmatrix}^T . \tag{44}$$

This operator has the property that $\|(\mathcal{H}\boldsymbol{\rho_A})_j\|_F = \|(\mathbf{H}\boldsymbol{\rho_A})_j\|_2$,[24] and so we can define the penalty function for this prior as

$$\lambda\Gamma\left((\mathbf{H}\boldsymbol{\rho_A})_j\right) = \lambda \sum_j \|(\mathbf{H}\boldsymbol{\rho_A})_j\|_2. \tag{45}$$

The corresponding subproblem can then be solved by block soft-thresholding:[25]

$$\begin{aligned} \mathrm{prox}_{\Gamma,\rho}(\mathbf{v}) &= \underset{\mathbf{z_2}}{\mathrm{argmin}} \ \lambda \sum_j \|(\mathbf{z_2})_j\|_2 + \frac{\rho}{2}\|\mathbf{v} - \mathbf{z_2}\|_2^2 && (46) \\ &= \max\left[\left(1 - \frac{\lambda/\rho}{\|(\mathbf{v})_j\|_2}\right)(\mathbf{v})_j, 0\right] && (47) \end{aligned}$$

Proximal operator for the indicator function. The proximal operator for the indicator function (Supplementary Equation (35)) is given by projection onto the convex set $\mathbb{R}_+$ with a projection operator $\Pi_{\mathbb{R}_+}$:

$$\begin{aligned} \mathrm{prox}_{\mathcal{I},\rho}(\mathbf{v}) &= \mathcal{I}_{\mathbb{R}_+}(\mathbf{z_3}) + \frac{\rho}{2}\|\mathbf{v} - \mathbf{z_3}\|_2^2 && (48) \\ &= \begin{cases} 0, & (\mathbf{v})_j < 0 \\ (\mathbf{v})_j, & (\mathbf{v})_j \geq 0. \end{cases} && (49) \end{aligned}$$

Supplementary Note 6: Simulated results

We evaluate our method in simulation for two scenes. In the first scene, we place a letter 'S' at a position 40 cm behind a 2.54-cm-thick scattering layer. The second scene contains two letters, an 'L' and 'T' placed at positions 30 cm and 40 cm behind the scattering layer. The measurements for both scenes are simulated for a grid of 32×32 confocal illuminated and imaged positions over a 60 cm $\times$ 60 cm square patch on the near side of the scattering layer. We generate the measurements using the full image formation model given by Supplementary Equation (9) and invert the model using the closed-form solution given by Supplementary Equation (24) with the *f–k* migration confocal inverse filter[14] and also using the iterative reconstruction method. We run the iterative reconstruction for 5000 iterations using the parameter values $\rho = 10^{-3}$ and $\lambda = 8 \times 10^{-5}$. The per-iteration time is approximately 33 ms for our GPU implementation running on an NVIDIA Titan X (Pascal).

Results for the closed-form solution for the 'S' scene applied to measurements with Poisson noise under varying signal levels and varying reduced scattering coefficients are shown in Supplementary Fig. 7. While the closed-form solution produces acceptable results in the highest signal regime, the performance degrades as the number of signal photons is reduced.

For these low signal levels, the iterative reconstruction method generally demonstrates improved performance compared to the closed-form method. We compare the iterative reconstruction method with gated imaging for the 'S' scene and the 'LT' scene for varying reduced scattering coefficients and signal photon levels in Supplementary Figures 8–13. A reference reconstruction is also shown for imaging through a thin diffuser with *f–k* migration.

For the comparison to gated imaging, we select the single time slice which produces the best qualitative result, showing the most hidden object structure. However, the time-gating approach produces results that are illegible and significantly more blurry than for confocal diffuse tomography. For the 'LT' scene with two letters at different distances, time gating can be used to partially recover the closest letter 'T', but not the more distant letter 'L' which is masked in the measurements by the tail of scattered light from the first letter. The iterative reconstructions are shown in Supplementary Figures 11–13 after compensating for radiometric falloff by applying a z-dependent scaling. This adjustment is applied uniformly across each of the results so that the more distant 'L' letter is more visible. Using confocal diffuse tomography, both letters are reconstructed and legible in all but the lowest signal level. All visualizations are shown using maximum intensity projection with a gamma correction of 1/3.

We note that the reconstruction quality generally improves as the reduced scattering coefficient is decreased (resulting in fewer scattering events), so that the most challenging simulated result is shown for a reduced scattering coefficient of 4.0 cm^{-1}. Quantitative results for the iterative reconstructions are also shown in Supplementary Table 1. We report peak signal-to-noise ratios (PSNR) calculated by comparing to a reference *f–k* Migration reconstruction for simulated measurements captured through a thin diffuser. In some cases, PSNR increases slightly with decreasing photon count; this appears to result from increased blur in the reconstruction improving the overlap between the reconstructed and reference shapes.

Supplementary Note 7: Additional captured results

We provide additional comparisons to different reconstruction methods applied to the captured data in Supplementary Fig. 14. Specifically, we compare CDT to time-gated imaging, in which we display a short time slice of the captured measurement in order to capture the first-arriving, minimally scattered photons. An additional comparison deconvolves the measurements with the diffusion kernel and displays the gated time slice. The deconvolution procedure corresponds to estimating the measurement response through a thin scattering layer (i.e., without diffusive scattering). In this case, time gating is still relatively ineffective because each scan point still collects photons from the entire surface of the hidden object. Thus, a single spatial location at each time slice may contain signal from multiple hidden object locations. We also report the photon counts of the captured measurements in Supplementary Table 2.

To evaluate the sensitivity of the method to the calibrated parameters of the scattering medium, we perform a qualitative perturbation analysis on the captured data. In Supplementary Fig. 15 we show reconstructions of the *Letters 'UT'* scene after perturbing the scattering layer thickness and the reduced scattering coefficient. The letters are legible in nearly all reconstructions, despite perturbing the parameters by greater than 30% of the nominal value.

Supplementary Note 8: Resolution limits

We derive axial and lateral resolution bounds in terms of the minimal resolvable distance of two scatterers separated by an axial distance Δz and lateral distance Δx using the full width at half maximum (FWHM) criterion as illustrated in Supplementary Fig. 16. A key factor in determining the resolution is the temporal response of light transmitted through the scattering layer. In our analysis, we neglect the contribution of laterally scattered light, so that the effect of the scatterer is primarily to broaden the incident light pulse in time. As with the approximated image formation model, this approximation holds when the spatial extent of the illuminated spot size at the far size of the scattering layer is small relative to the axial distance to the scatterer.

The temporal response of light which propagates through the scattering layer and back is the dominant consideration in determining the resolution. While practical systems also use detectors and illumination sources with a finite temporal response, in this analysis we assume that this is negligible relative to the response of the scattering layer. We denote the full width half maximum of the temporal response as Δt. For scattering in the diffusive regime, the temporal spread of a pulse of light can be approximated using the diffusive traversal time $\Delta t_{\mathrm{d}} = \frac{z_{\mathrm{d}}^2}{6Dc}$ which is the typical time it takes for a photon to diffuse one way through the medium.[26] If we take the temporal spread for two-way propagation to be approximately twice the diffusive traversal time $\Delta t = 2\Delta t_{\mathrm{d}}$, we can use this value to consider the axial and lateral resolution bounds.

As depicted in Supplementary Fig. 16, the spatial locations of two point scatterers, $\mathbf{x_1}$ and $\mathbf{x_2}$ can be resolved from the time-resolved measurements if the distance between them satisfies the following relationship

$$\mathrm{abs}\left(\|\mathbf{r} - \mathbf{x_1}\| - \|\mathbf{r} - \mathbf{x_2}\|\right) \geq \frac{c\Delta t}{2}. \tag{50}$$

From this expression, the axial resolution can readily be defined as $\Delta z \geq \frac{c\Delta t}{2}$.

For the lateral resolution, we follow the derivation of O'Toole et al.[13] and assume that the lateral size over which the scattering layer is sampled is much greater then the lateral distance Δx between $\mathbf{x_1}$ and $\mathbf{x_2}$. Under this assumption, we have that

$$\frac{c\Delta t}{2} \approx \Delta x \frac{w}{\sqrt{w^2 + z^2}}, \tag{51}$$

where w is the lateral distance between the scatterers and the sampled position on the scattering layer and z is the axial distance to the scatterers as depicted in Supplementary Fig. 16. Then, the lateral resolution is given as

$$\Delta x \geq \frac{c\sqrt{w^2 + z^2}}{2w}\Delta t. \tag{52}$$

The axial resolution is constant with increasing depth; however, the lateral resolution decays linearly with increasing depth. The sampled area on the scattering layer also affects the resolution. Intuitively, the sampled area acts as an imaging aperture and, as in other imaging modalities, the greater the sampled aperture size (or sampled area), the better the lateral resolution.

For our hardware system, we measure Δt by placing a retroreflective target behind the scattering medium and capturing the response at a single confocal scan point. We find that the FWHM of the twice-diffused temporal response is approximately 640 ps, as shown in Supplementary Fig. 17, which corresponds to an axial resolution of 8.6 cm and compares well to the diffusive traversal time $2\Delta t_\text{d} \approx 632$ ps. The lateral resolution is measured experimentally using a retroreflective resolution target with three groups of stripe patterns separated by 30 cm, 20 cm, or 10 cm from the center of one stripe to another. The target is captured at a distance of 50 cm and 70 cm behind the scattering medium where the predicted lateral resolutions are 15 cm and 19 cm, respectively. In Supplementary Fig. 17, this corresponds to the smallest pattern being just outside the resolution limit at 50 cm distance and the middle pattern being just resolvable at 70 cm distance, which is observed in practice.

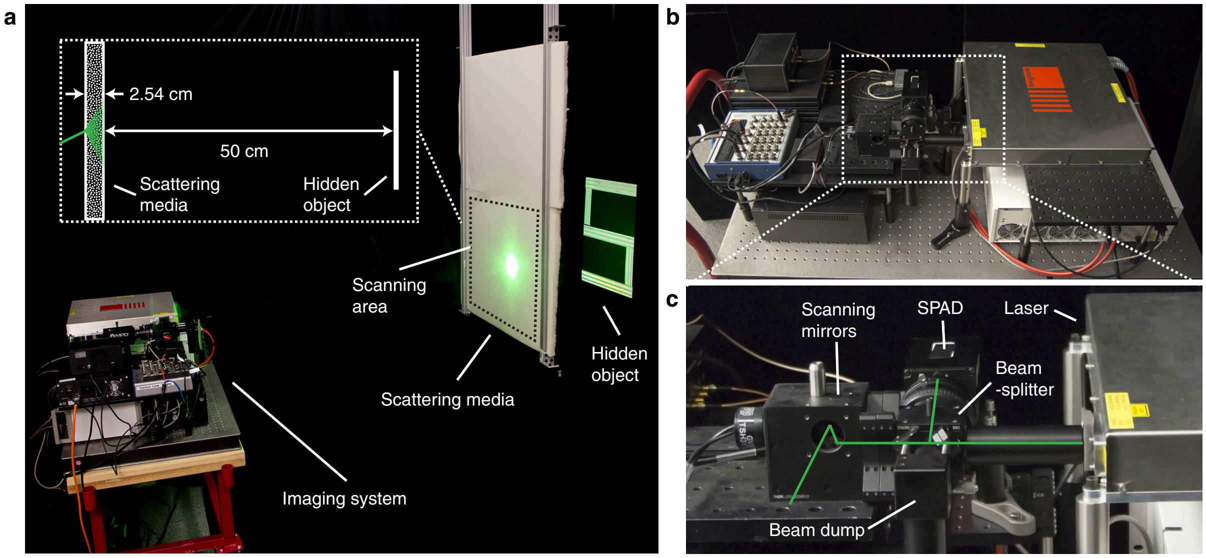

Supplementary Fig. 1 Hardware prototype. a Measurements are captured using the hardware prototype to scan points on the surface of the scattering medium. **b** The primary components of the hardware prototype consist of a pulsed laser which shares an optical path through a beam splitter with a single-pixel SPAD detector to image and illuminate positions scanned using a two-axis galvanometer. **c** Optical paths are illustrated by the green line.

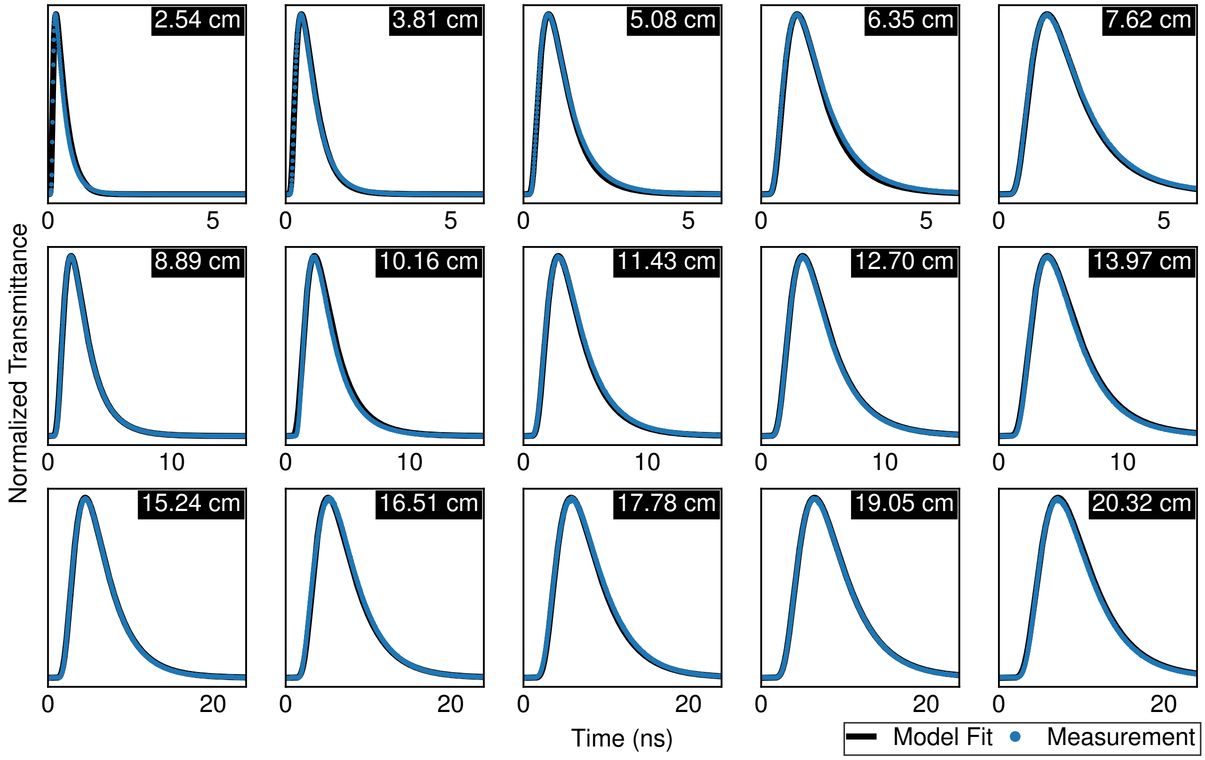

Supplementary Fig. 2 Calibration of scattering and absorption parameters. The scattering and absorption parameters of the scattering medium are characterized by fitting the predicted diffusion profile (black line) to a time-resolved measurement (blue dots) of a short laser pulse propagating through polyurethane foam of varying thicknesses (denoted in upper right corner of each plot). Plots are shown for the parameter values that provide the best fit to the data: $\mu_a = 5.29 \times 10^{-3}\,\text{cm}^{-1}$ and $\mu_s' = 2.62\,\text{cm}^{-1}$.

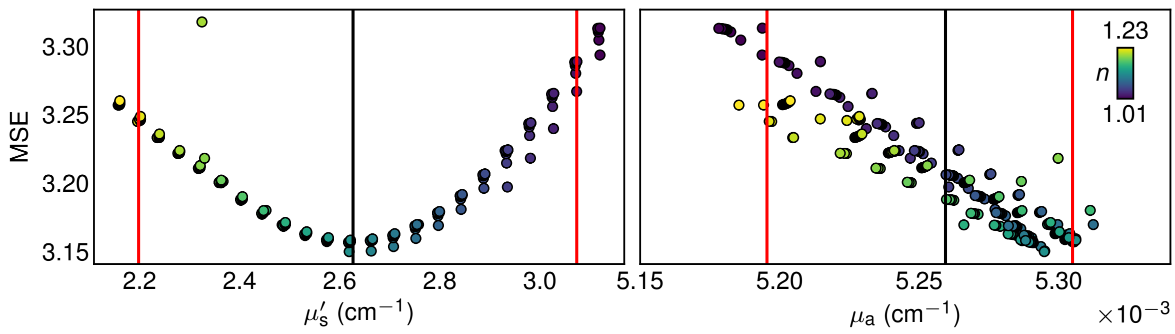

Supplementary Fig. 3 Parameter confidence intervals. To quantify uncertainty in the calibrated scattering parameters, we optimize for the parameters that best fit the data given a range of possible refractive index values, n, for the scattering medium. We run 253 optimizations for 23 refractive index values ranging from 1.01 to 1.23. For each refractive index value, we optimize for 11 different initializations of the scattering coefficient, from 2.0 cm^{-1} to 3.0 cm^{-1}. The y-axis represents the mean squared error (MSE) of the model fit across all 15 captured measurements, and color indicates the refractive index used in the optimization. The mean (black line) and confidence intervals (red lines) of the optimized values are $\mu_s' = 2.62 \pm 0.43\,\text{cm}^{-1}$ and $\mu_a = 5.26 \times 10^{-3} \pm 5.5 \times 10^{-5}\,\text{cm}^{-1}$. Here, the confidence interval indicates the range containing 95% of the parameter values.

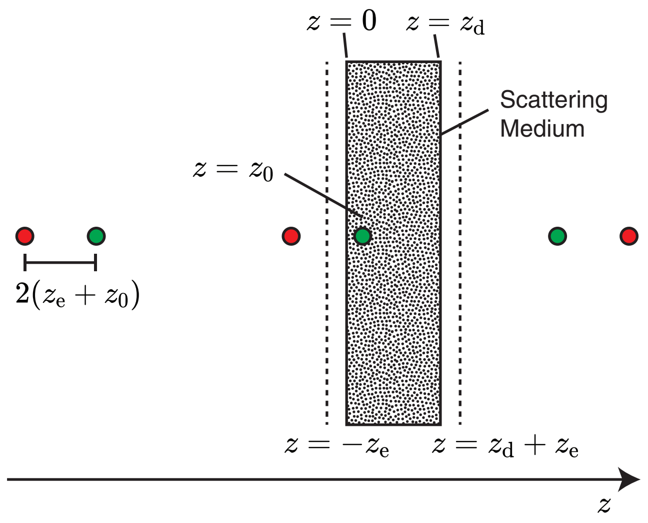

Supplementary Fig. 4 Positions of dipole sources for slab geometry. The solution of the diffusion equation for the slab geometry places an infinite number of positive (green) and negative (red) dipole sources such that the diffusive intensity is zero at an extrapolated surface (dashed line) that is displaced from either side of the scattering medium. The first three dipole sources are shown.

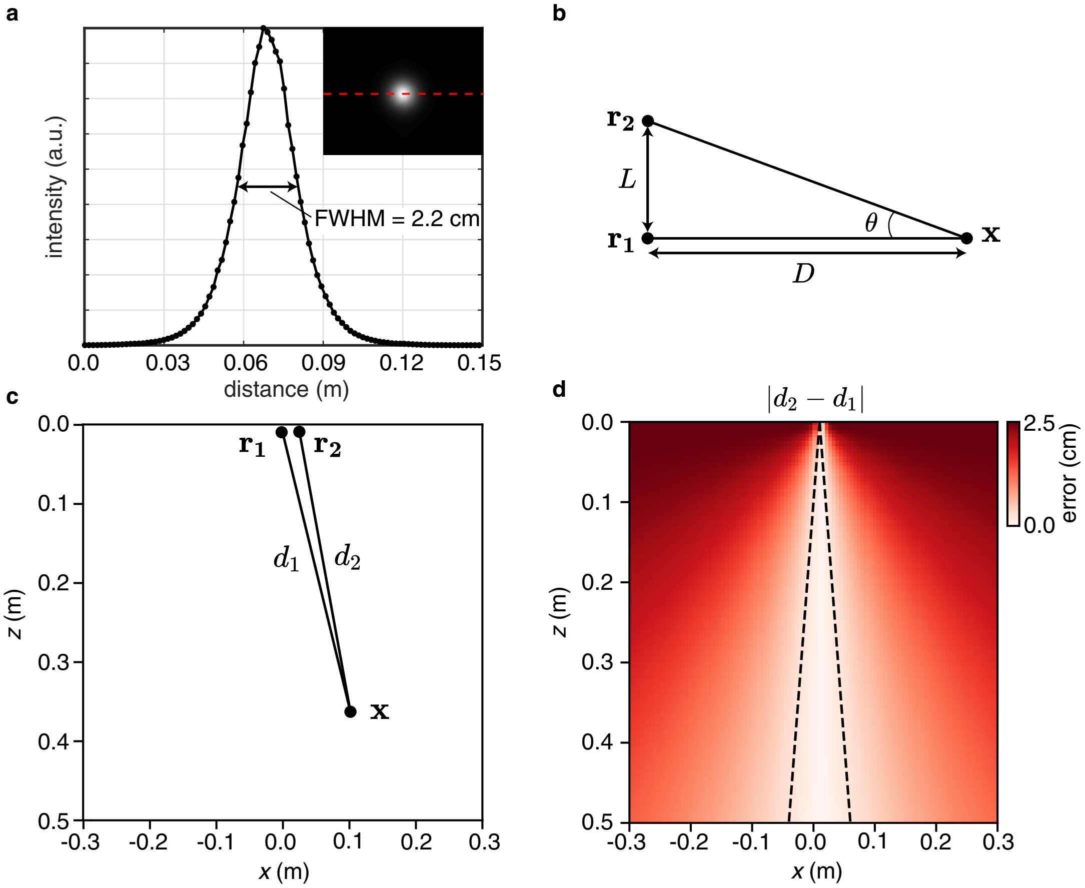

Supplementary Fig. 5 Forward model approximation error. **a** The full width at half maximum (FWHM) of the measured spot size on the far side of the scattering medium is plotted for a cross section (shown, inset) and is approximately 2.2 cm. **b** Using the spot size, the error of the approximate forward model can be expressed as $L^2/(2H)$ using a paraxial approximation for small angles of θ. **c–d** The exact error given by the difference between $d_1 = \|\mathbf{r_1} - \mathbf{x}\|$ and $d_2 = \|\mathbf{r_2} - \mathbf{x}\|$, illustrated for an example location of a point scatterer $\mathbf{x}$ and $\mathbf{r_1}$ and $\mathbf{r_2}$ separated by 2.5 cm. **d** The error follows the paraxial approximation within the dotted lines, and reaches a maximum value of 2.5 cm at the greatest incidence angles.

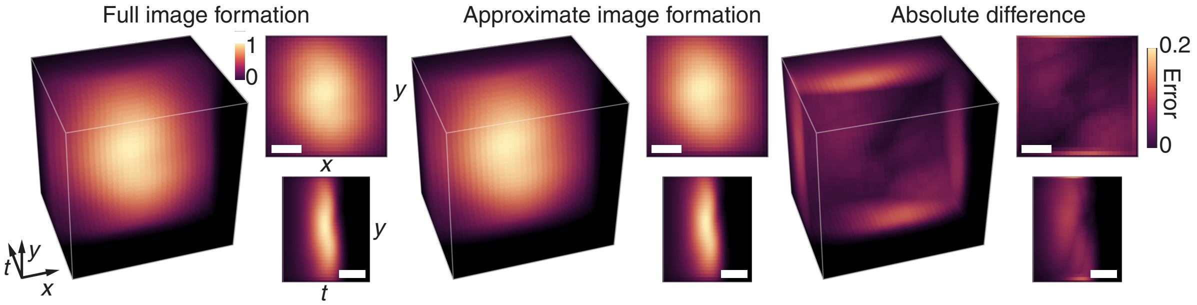

Supplementary Fig. 6 Comparison of forward models. Maximum intensity projections for the full forward model (left) and approximate forward model (middle) are visualized with maximum intensity projections for a simulated letter "S" scene with a reduced scattering coefficient of $\mu_s' = 3.0\,\text{cm}^{-1}$. The measurements are normalized so that the maximum value is one, and the absolute difference is also shown (right, arbitrary unit). The approximate measurements show good agreement with the full forward model, with the largest differences occurring around volume boundaries. The mean absolute error is 0.007. Scale bars indicate 15 cm or 1 ns.

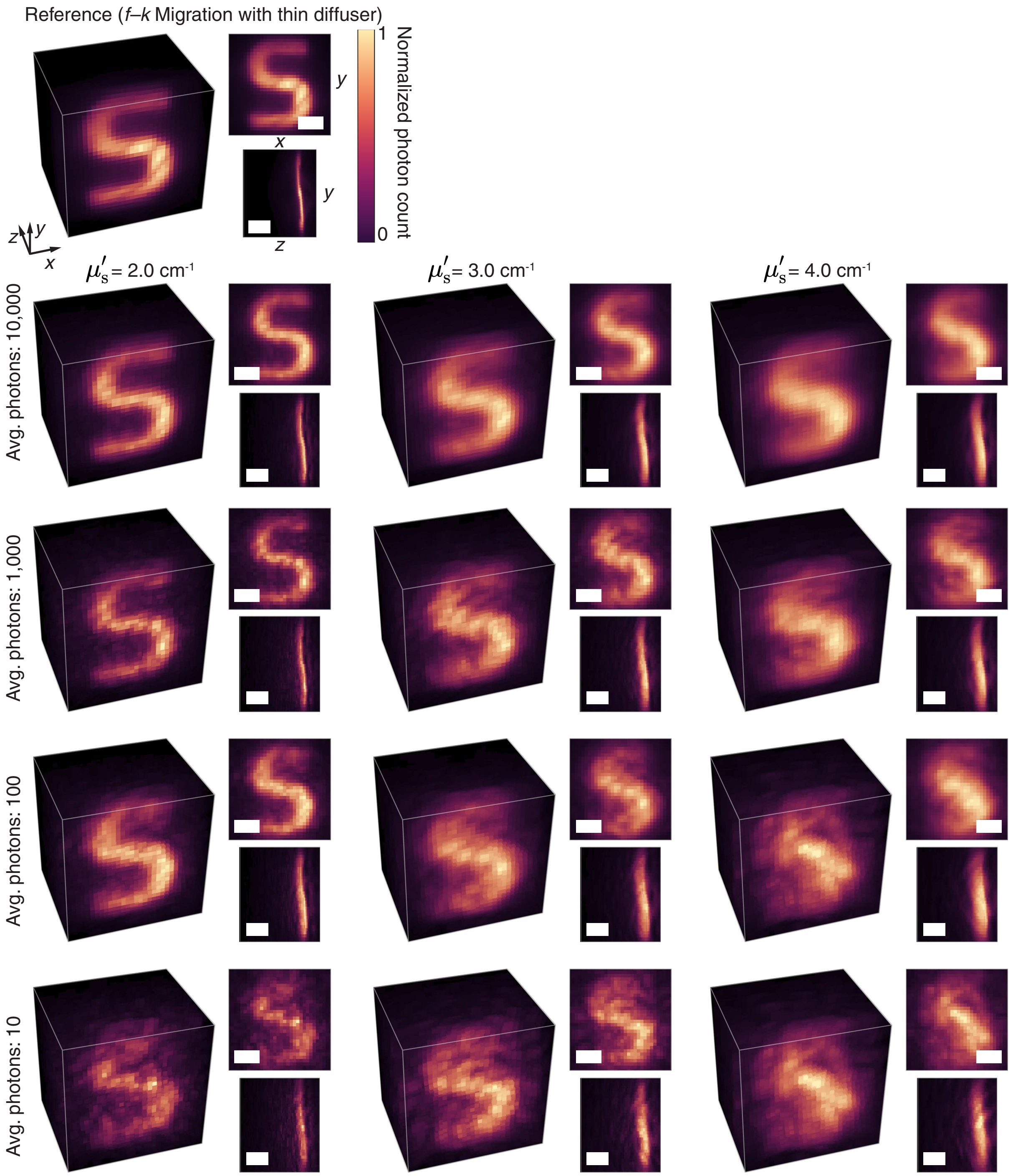

Supplementary Fig. 7 Closed-form reconstruction for simulated letter 'S'. A reference reconstruction is computed (first row) for imaging through a thin diffuser with *f*–*k* migration.[14] The closed-form reconstruction is applied to measurements with varying numbers of average signal photons and a range of reduced scattering coefficient values. The simulated absorption coefficient is $0.5\,\text{cm}^{-1}$. The dimensions of the reconstructed volumes are $32 \times 32 \times 64$ voxels and $0.6 \times 0.6 \times 0.5$ m along the x, y, and z directions, respectively. All scale bars indicate 15 cm.

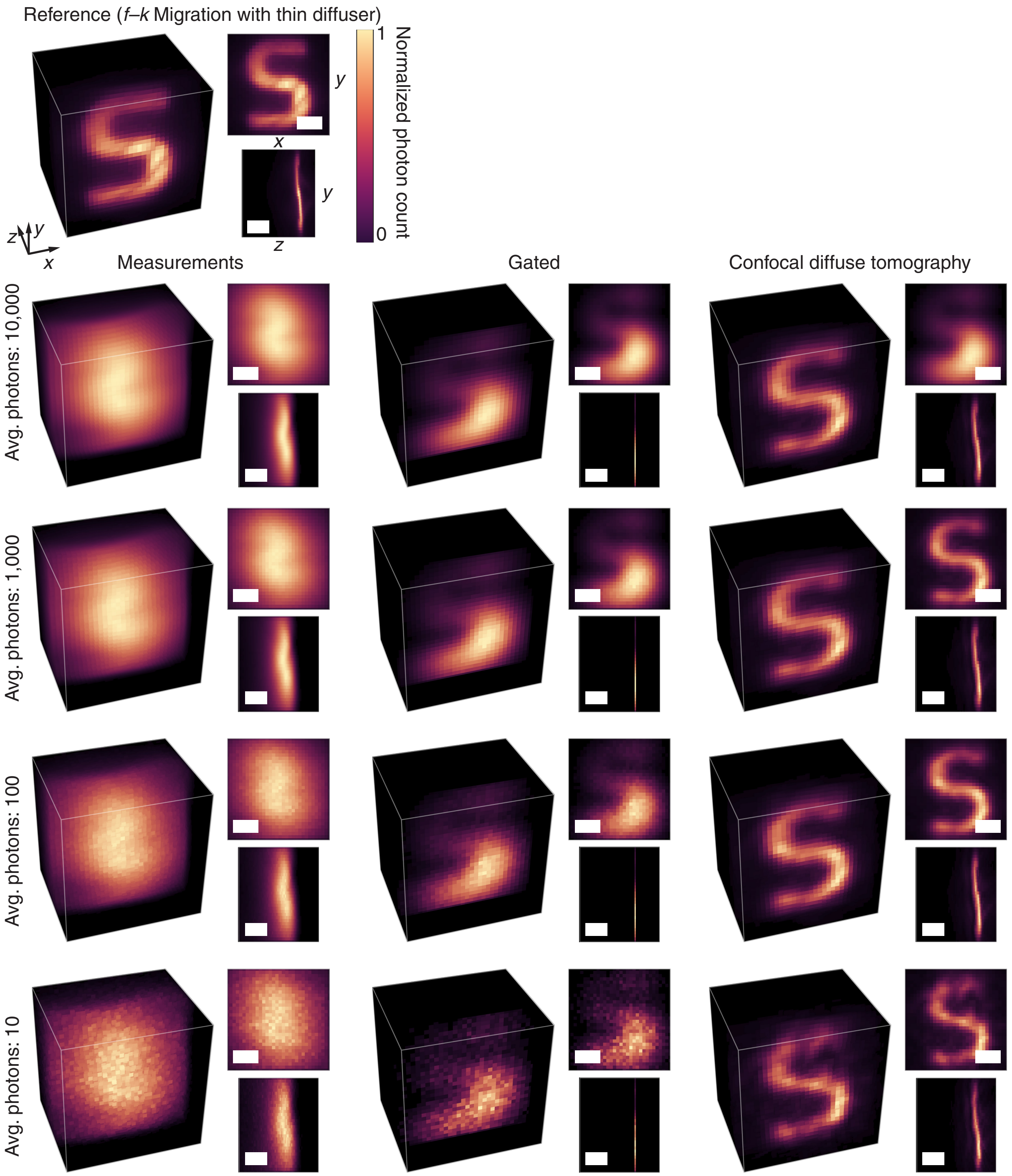

Supplementary Fig. 8 Simulated letter 'S' for $\mu_s' = 2.0\,\text{cm}^{-1}$ and $\mu_a = 0.5\,\text{cm}^{-1}$. A reference reconstruction is computed (first row) for imaging through a thin diffuser with *f*–*k* migration.[14] From the measurement volumes, a gated reconstruction from a single time slice and reconstructions using confocal diffuse tomography are visualized using maximum intensity projections for varying numbers of average photons per voxel. The dimensions of the reconstructed volumes are $32 \times 32 \times 64$ voxels and $0.6 \times 0.6 \times 0.5$ m along the x, y, and z directions, respectively. All scale bars indicate 15 cm or 1 ns.

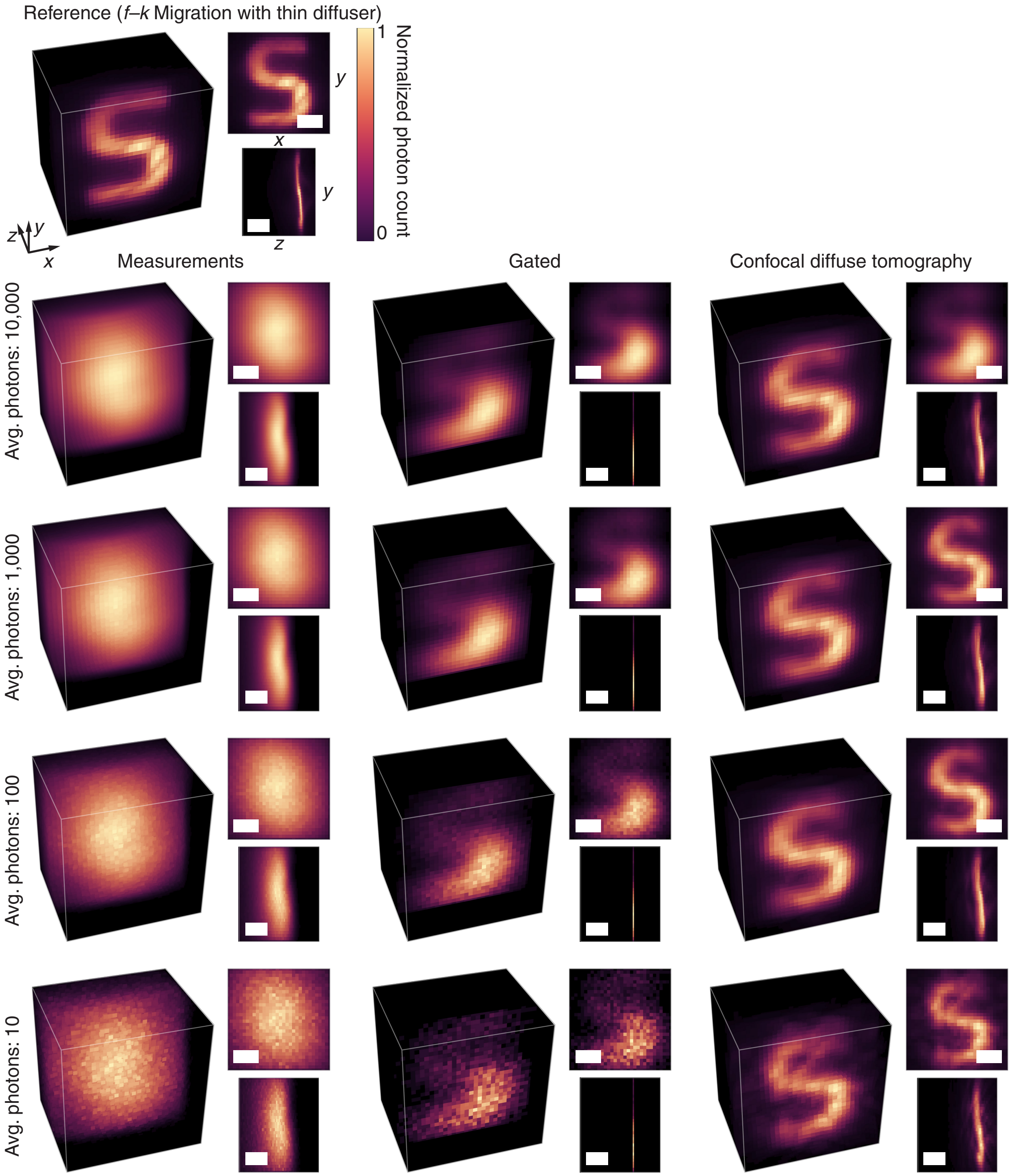

Supplementary Fig. 9 Simulated letter 'S' for $\mu_s' = 3.0\,\text{cm}^{-1}$ and $\mu_a = 0.5\,\text{cm}^{-1}$. A reference reconstruction is computed (first row) for imaging through a thin diffuser with *f*–*k* migration.[14] From the measurement volumes, a gated reconstruction from a single time slice and reconstructions using confocal diffuse tomography are visualized using maximum intensity projections for varying numbers of average photons per voxel. The dimensions of the reconstructed volumes are $32 \times 32 \times 64$ voxels and $0.6 \times 0.6 \times 0.5$ m along the x, y, and z directions, respectively. All scale bars indicate 15 cm or 1 ns.

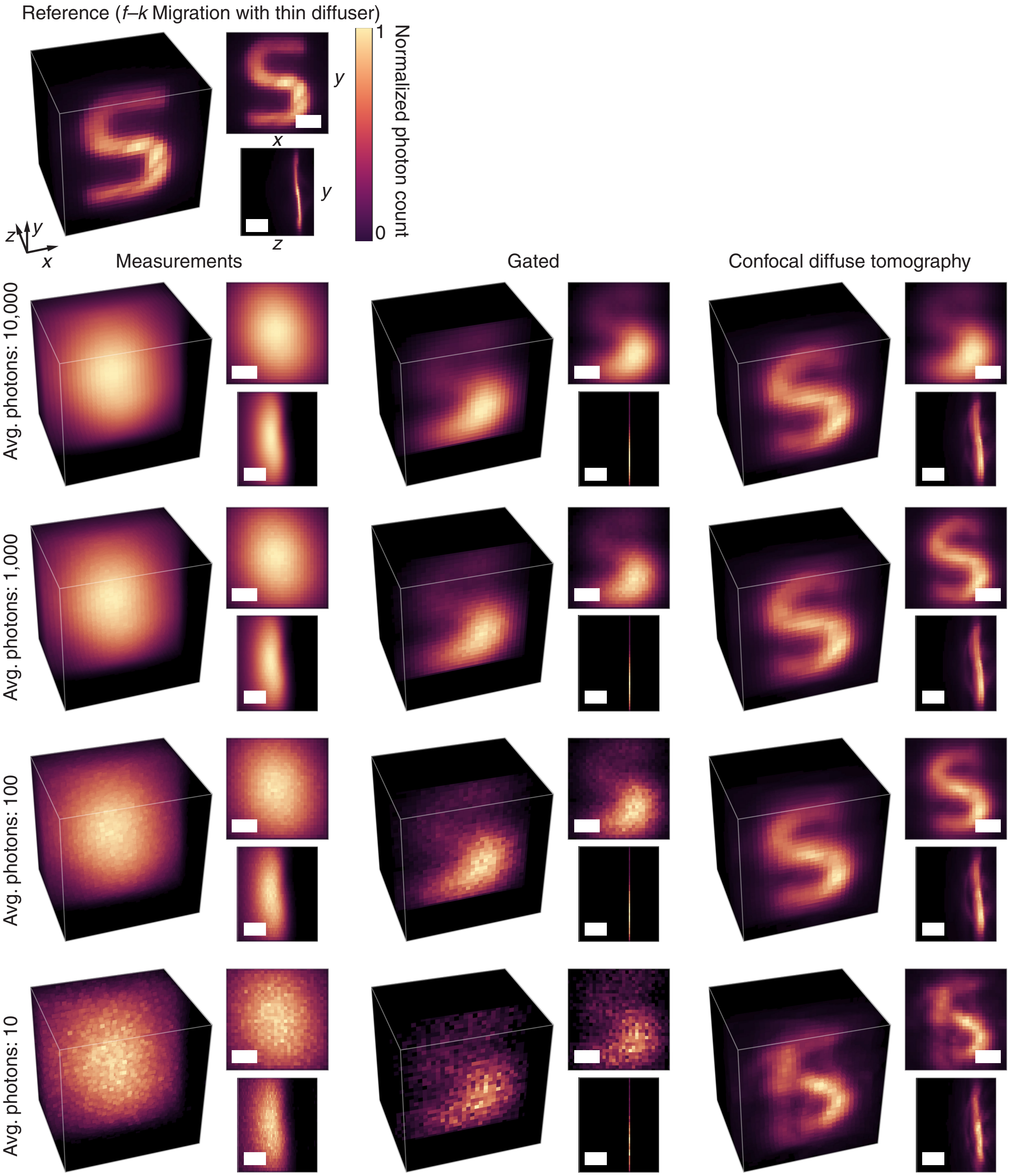

Supplementary Fig. 10 Simulated letter 'S' for $\mu_s' = 4.0\,\mathrm{cm}^{-1}$ and $\mu_a = 0.5\,\mathrm{cm}^{-1}$. A reference reconstruction is computed (first row) for imaging through a thin diffuser with *f–k* migration.[14] From the measurement volumes, a gated reconstruction from a single time slice and reconstructions using confocal diffuse tomography are visualized using maximum intensity projections for varying numbers of average photons per voxel. The dimensions of the reconstructed volumes are $32 \times 32 \times 64$ voxels and $0.6 \times 0.6 \times 0.5$ m along the x, y, and z directions, respectively. All scale bars indicate 15 cm or 1 ns.

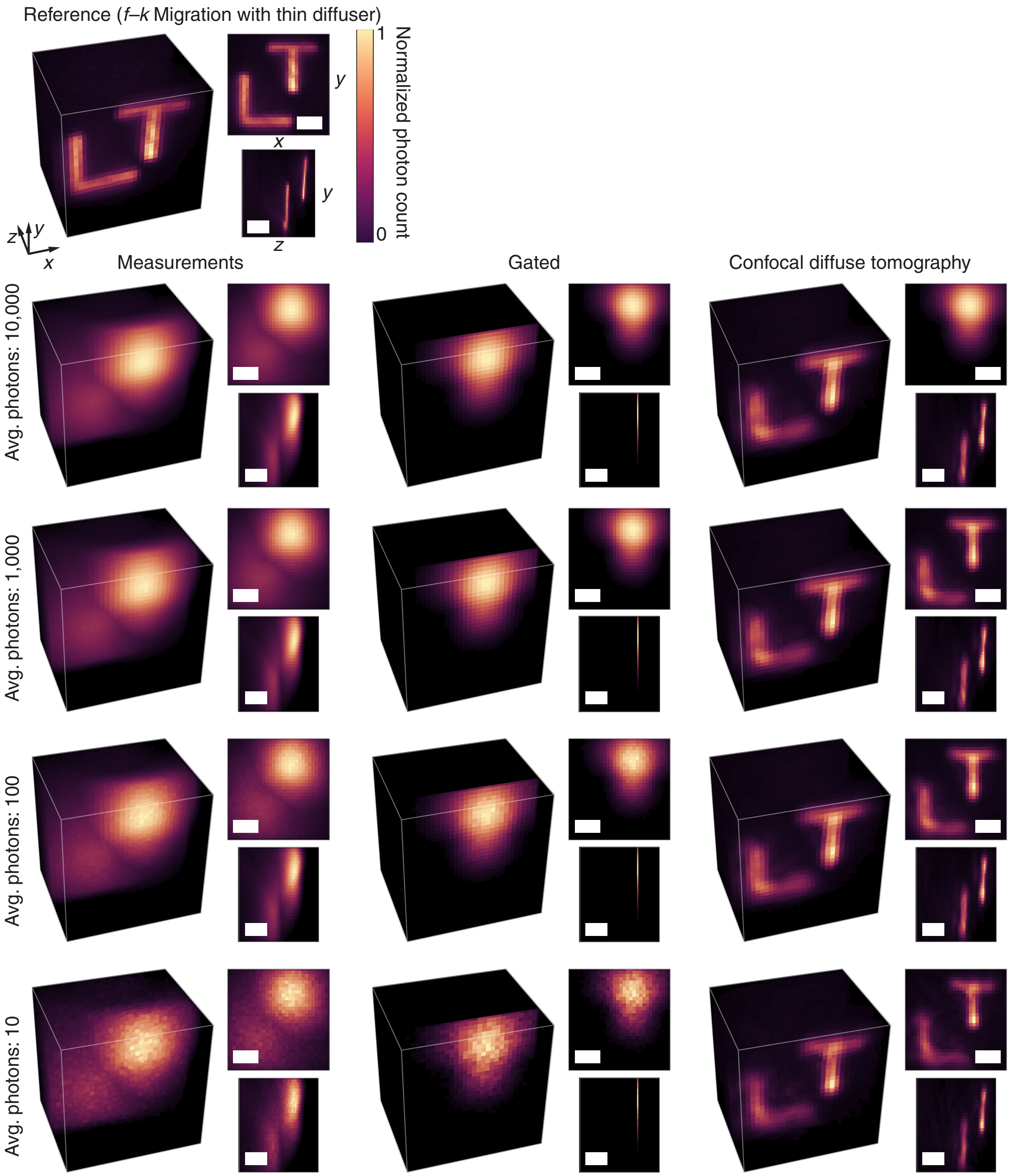

Supplementary Fig. 11 Simulated letters 'LT' for $\mu_s' = 2.0\,\mathrm{cm}^{-1}$ and $\mu_a = 0.5\,\mathrm{cm}^{-1}$. A reference reconstruction is computed (first row) for imaging through a thin diffuser with *f*–*k* migration.[14] From the measurement volumes, a gated reconstruction from a single time slice and reconstructions using confocal diffuse tomography are visualized using maximum intensity projections for varying numbers of average photons per voxel. The dimensions of the reconstructed volumes are $32 \times 32 \times 64$ voxels and $0.6 \times 0.6 \times 0.5$ m along the x, y, and z directions, respectively. A depth-dependent scaling is applied to account for radiometric falloff. All scale bars indicate 15 cm or 1 ns.

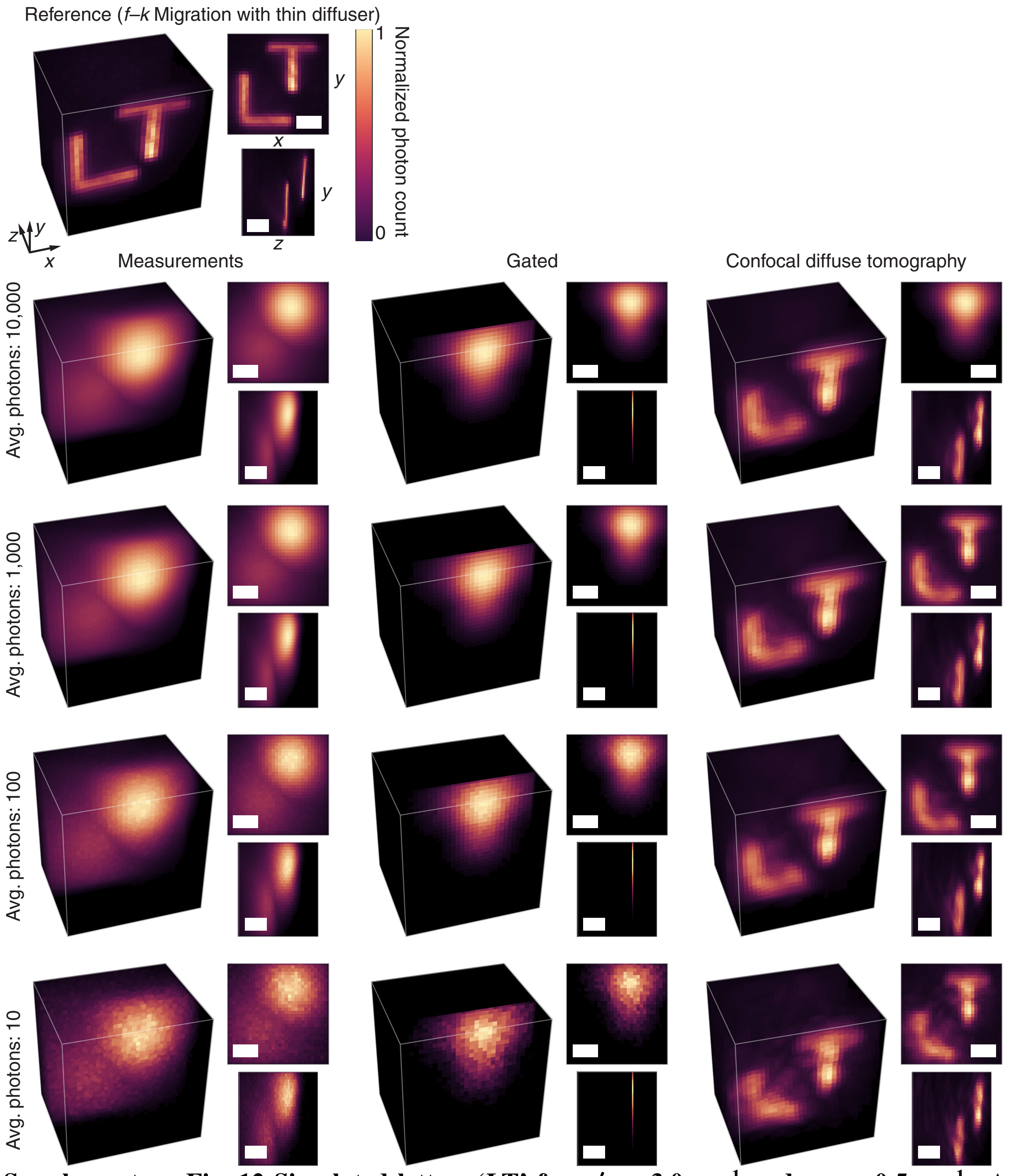

Supplementary Fig. 12 Simulated letters 'LT' for $\mu_s' = 3.0\,\mathrm{cm}^{-1}$ and $\mu_a = 0.5\,\mathrm{cm}^{-1}$. A reference reconstruction is computed (first row) for imaging through a thin diffuser with f–k migration.[14] From the measurement volumes, a gated reconstruction from a single time slice and reconstructions using confocal diffuse tomography are visualized using maximum intensity projections for varying numbers of average photons per voxel. The dimensions of the reconstructed volumes are $32 \times 32 \times 64$ voxels and $0.6 \times 0.6 \times 0.5$ m along the x, y, and z directions, respectively. A depth-dependent scaling is applied to account for radiometric falloff. All scale bars indicate 15 cm or 1 ns.

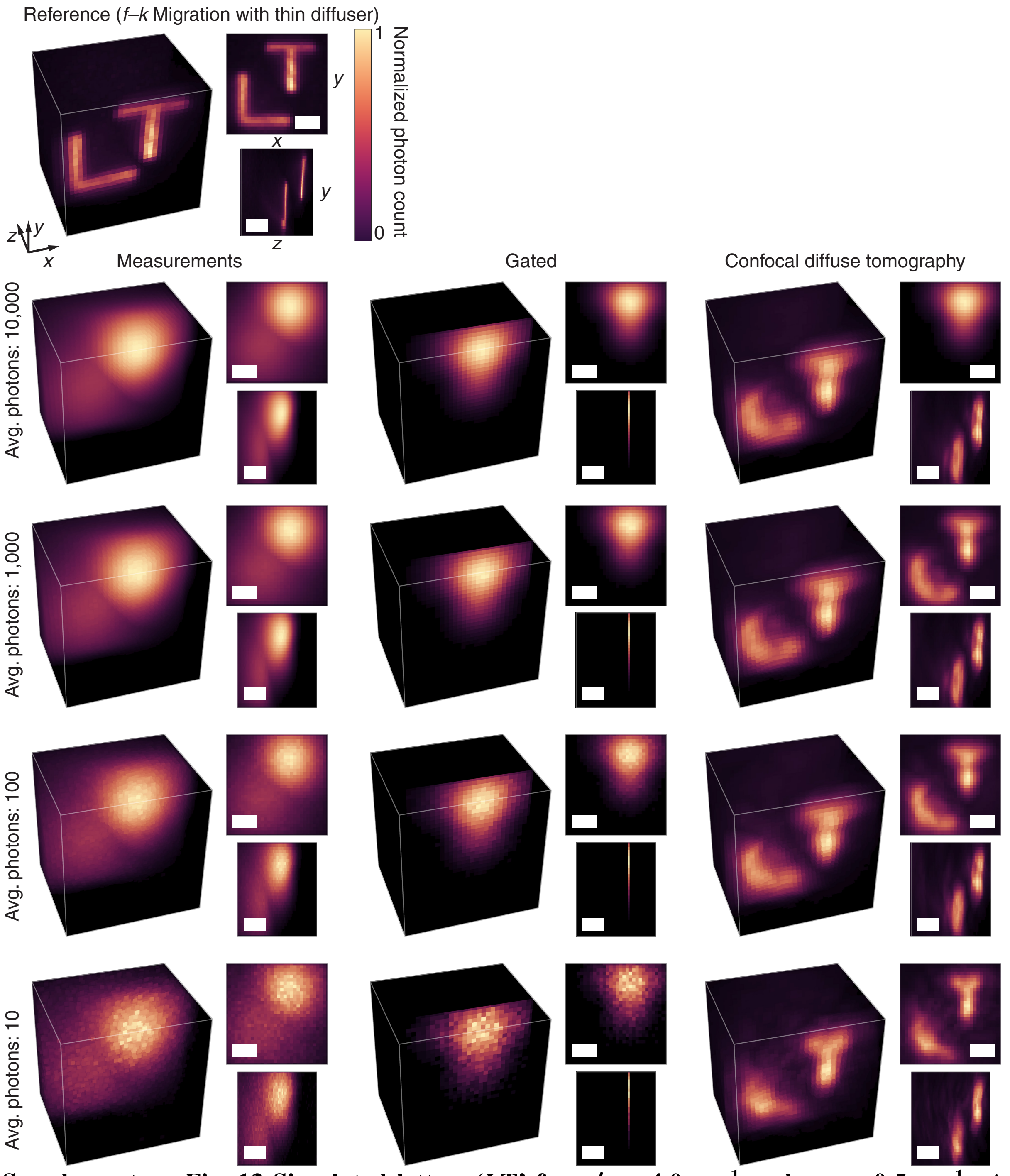

Supplementary Fig. 13 Simulated letters 'LT' for $\mu_s' = 4.0\,\text{cm}^{-1}$ and $\mu_a = 0.5\,\text{cm}^{-1}$. A reference reconstruction is computed (first row) for imaging through a thin diffuser with *f*–*k* migration.[14] From the measurement volumes, a gated reconstruction from a single time slice and reconstructions using confocal diffuse tomography are visualized using maximum intensity projections for varying numbers of average photons per voxel. The dimensions of the reconstructed volumes are $32 \times 32 \times 64$ voxels and $0.6 \times 0.6 \times 0.5$ m along the x, y, and z directions, respectively. A depth-dependent scaling is applied to account for radiometric falloff. All scale bars indicate 15 cm or 1 ns.

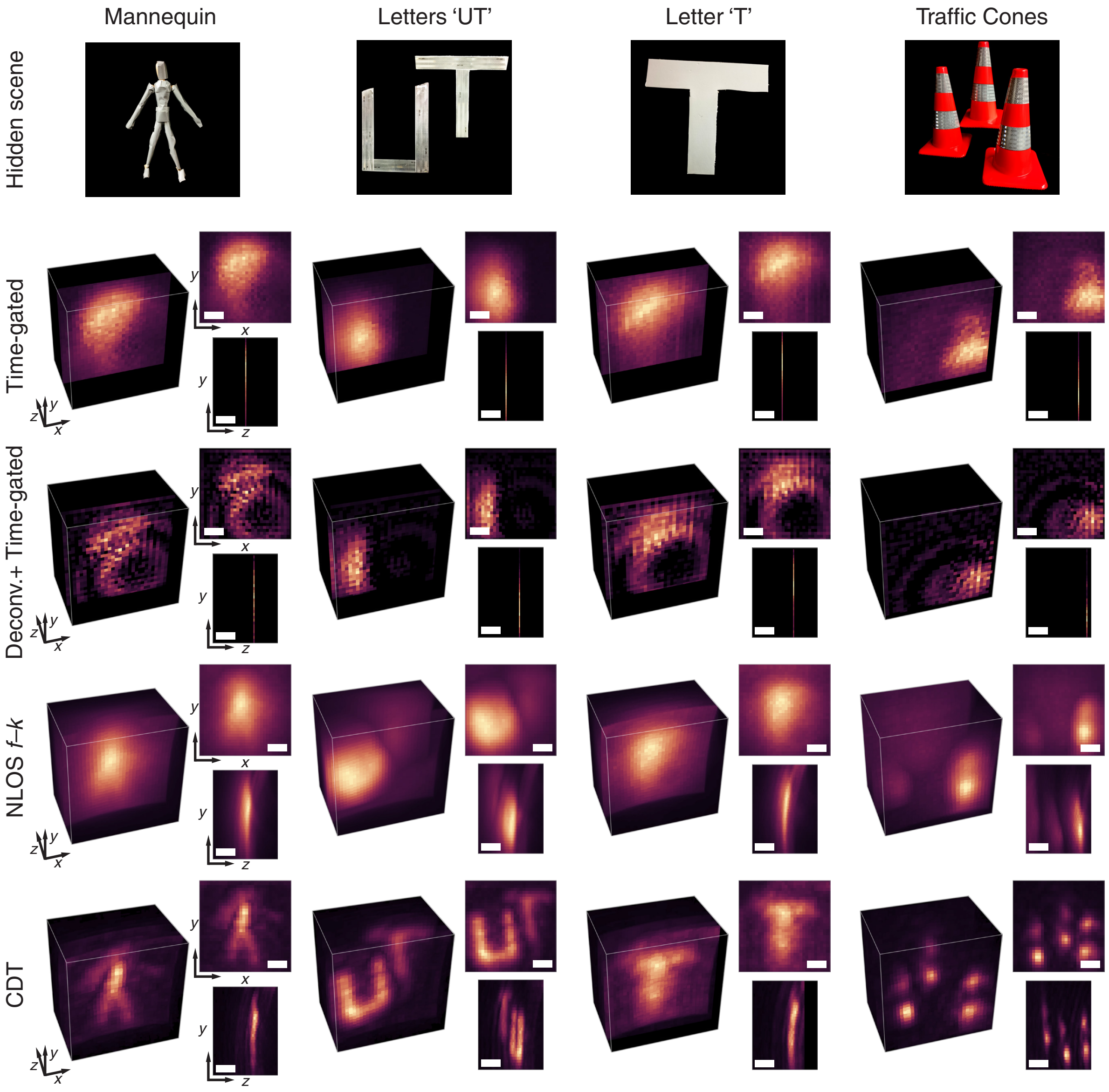

Supplementary Fig. 14 Comparison of reconstruction methods. A hidden scene consists of a retroreflective mannequin (first column), and the scene is reconstructed using time gating, deconvolution and time gating, *f*–*k* migration for non-line-of-sight (NLOS) imaging,[14] and CDT (rows, top to bottom). Scene photos and reconstructions are also shown for two retroreflective letters, a diffuse letter, and a group of traffic cones. We apply a depth-dependent scaling to the traffic cone visualization to account for radiometric falloff. Scalebars indicate 15 cm or 1 ns, and a gamma of 1/3 is applied to all maximum intensity projection visualizations.

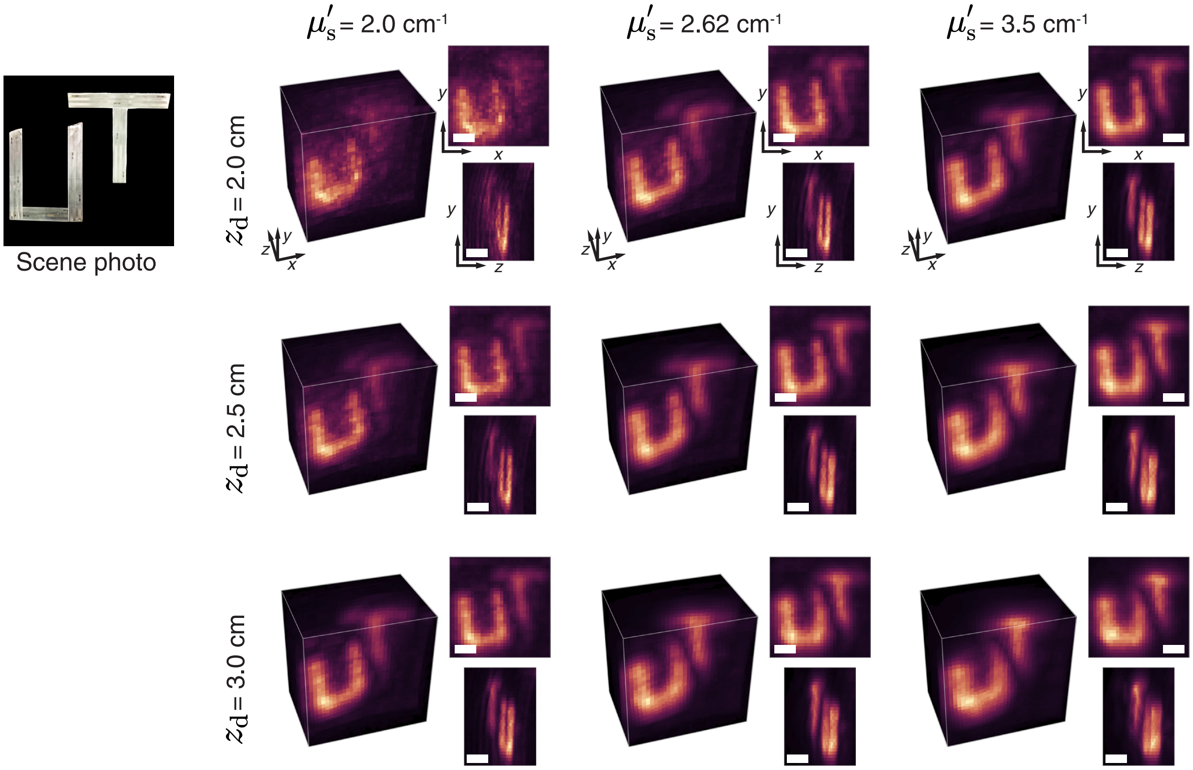

Supplementary Fig. 15 Perturbation analysis. The sensitivity of the reconstruction to the calibrated scattering medium parameters is evaluated by reconstructing the *Letters 'UT'* scene using perturbed reduced scattering coefficients (μ_s') and scattering layer thicknesses (z_d). The center reconstruction ($\mu_s' = 2.62$ cm^{-1}, $z_d = 2.5$ cm) uses the nominal values.

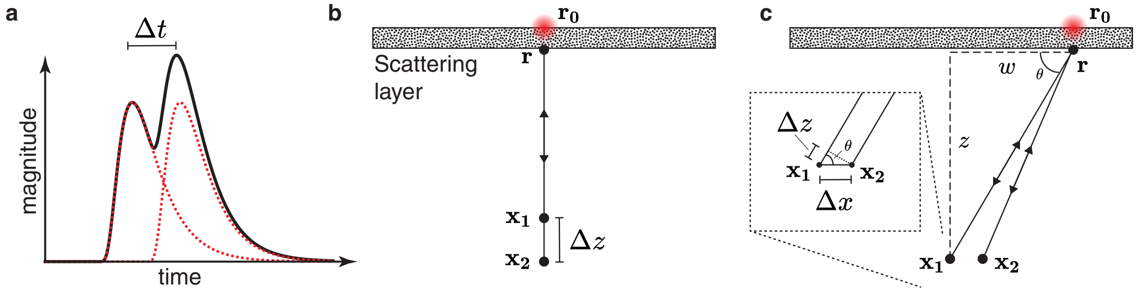

Supplementary Fig. 16 Illustration of resolution analysis. **a** The resolution is considered using the full width at half maximum (FWHM) criterion, such that two point scatterers are just resolvable when their point spread functions are separated by the FWHM. **b** The axial resolution Δz for a short illumination pulse which propagates through the scattering layer from a point $\mathbf{r}$ to $\mathbf{r_0}$, and is backscattered by two point scatterers $\mathbf{x_1}$ and $\mathbf{x_2}$ is directly proportional to the FWHM. **c** The lateral resolution Δx is derived following O'Toole et al.[13] and can be expressed as a function of the axial distance z, the lateral distance w, the angle θ, and the FWHM.

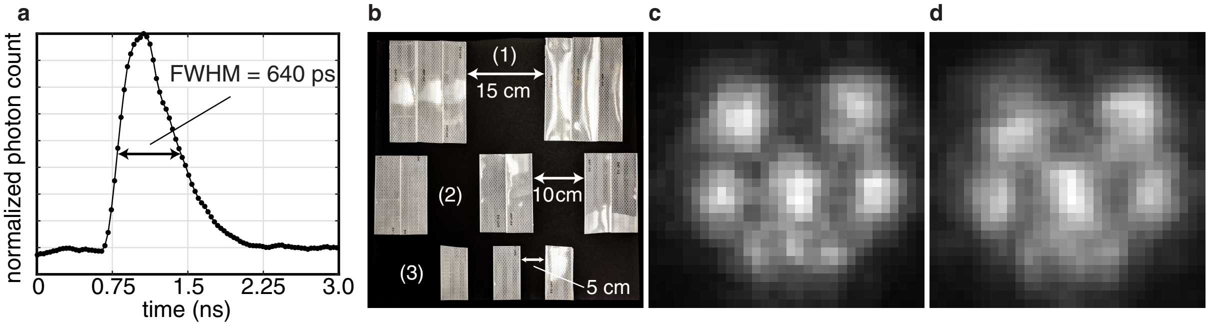

Supplementary Fig. 17 Measured resolution. **a** The axial resolution of the system is measured by capturing the temporal response of a retroreflective target placed behind the scattering medium and acquiring a confocal measurement. The FWHM of the resulting measurement represents the system temporal resolution, Δt =640 ps, giving a theoretical axial resolution of 8.6 cm. **b** The lateral resolution is estimated by capturing a retroreflective resolution chart with three groups of targets whose centers are separated by 30 cm, 20 cm, and 10 cm. Measurements are captured for a target distance of 50 cm (**c**) and 70 cm (**d**) behind the scattering medium. The theoretical lateral resolution predicts that group (3) is just outside the resolution limit at 50 cm and group (2) is just resolvable at 70 cm, which is observed in practice.

	Avg. photons	PSNR (dB) $\mu_s' = 2.0\,\mathrm{cm}^{-1}$	$\mu_s' = 3.0\,\mathrm{cm}^{-1}$	$\mu_s' = 4.0\,\mathrm{cm}^{-1}$
**Letter ‘S’**	10000	28.11	28.61	25.69
	1000	28.10	28.88	25.62
	100	27.87	28.50	26.02
	10	27.64	28.88	27.66
**Letters ‘LT’**	10000	32.67	29.98	27.94
	1000	32.75	29.80	28.26
	100	32.90	30.29	27.79
	10	32.52	31.02	28.74

Supplementary Table 1 Peak signal-to-noise ratio (PSNR) values for iterative reconstruction of ‘S’ and ‘LT’ scenes. To calculate PSNR, the iterative reconstructions are compared with a reference *f–k* Migration reconstruction for simulated measurements captured through a thin diffuser. Reconstruction values are normalized to the range $[0, 1]$ prior to calculating PSNR.

Experiment name	Retroreflective	Exposure	Photon counts
Letter ‘S’	Yes	60 ms	1.9×10^4
Mannequin	Yes	700 ms	4.5×10^4
Letters ‘UT’	Yes	600 ms	1.5×10^5
Letter ‘T’	No	3500 ms	3.6×10^4
Traffic Cones	Yes	390 ms	2.9×10^4
Resolution Chart (50 cm)	Yes	312 ms	4.8×10^4
Resolution Chart (70 cm)	Yes	312 ms	3.9×10^4

Supplementary Table 2 Experimental details for captured results. The exposure per scan position and average number of photon counts per histogram across all spatial samples is reported for captured results shown in Fig. 2 and Fig. 3 of the main paper and Supplementary Figure 14, Supplementary Figure 15, and Supplementary Figure 17.

Supplementary References

[1] Paszke, A. *et al.* Pytorch: An imperative style, high-performance deep learning library. In *Proc. NeurIPS*, 8026–8037 (2019).

[2] Reddi, S. J., Kale, S. & Kumar, S. On the convergence of Adam and beyond. In *Proc. ICLR* (2018).

[3] Contini, D., Martelli, F. & Zaccanti, G. Photon migration through a turbid slab described by a model based on diffusion approximation. I. Theory. *Appl. Opt.* **36**, 4587–4599 (1997).

[4] Zhu, J., Pine, D. & Weitz, D. Internal reflection of diffusive light in random media. *Phys. Rev. A* **44**, 3948 (1991).

[5] Zhang, H., Fang, W.-Z., Li, Y.-M. & Tao, W.-Q. Experimental study of the thermal conductivity of polyurethane foams. *Appl. Therm. Eng.* **115**, 528–538 (2017).

[6] Patterson, M. S., Chance, B. & Wilson, B. C. Time resolved reflectance and transmittance for the noninvasive measurement of tissue optical properties. *Appl. Opt.* **28**, 2331–2336 (1989).

[7] Farrell, T. J., Patterson, M. S. & Wilson, B. A diffusion theory model of spatially resolved, steady-state diffuse reflectance for the noninvasive determination of tissue optical properties in vivo. *Med. Phys.* **19**, 879–888 (1992).

[8] Haskell, R. C. *et al.* Boundary conditions for the diffusion equation in radiative transfer. *JOSA A* **11**, 2727–2741 (1994).

[9] Faccio, D., Velten, A. & Wetzstein, G. Non-line-of-sight imaging. *Nat. Rev. Phys.* **2**, 318–327 (2020).

[10] Xin, S. *et al.* A theory of Fermat paths for non-line-of-sight shape reconstruction. In *Proc. CVPR*, 6800–6809 (2019).

[11] Dutre, P., Bekaert, P. & Bala, K. *Advanced global illumination* (AK Peters/CRC Press, 2006).

[12] Pharr, M., Jakob, W. & Humphreys, G. *Physically based rendering: From theory to implementation* (Morgan Kaufmann, 2016).

[13] O'Toole, M., Lindell, D. B. & Wetzstein, G. Confocal non-line-of-sight imaging based on the light-cone transform. *Nature* **555**, 338–341 (2018).

[14] Lindell, D. B., Wetzstein, G. & O'Toole, M. Wave-based non-line-of-sight imaging using fast f–k migration. *ACM Trans. Graph.* **38**, 1–13 (2019).

[15] Freund, I. Looking through walls and around corners. *Physica A* **168**, 49–65 (1990).

[16] Stolt, R. H. Migration by Fourier transform. *Geophysics* **43**, 23–48 (1978).

[17] Cafforio, C., Prati, C. & Rocca, F. SAR data focusing using seismic migration techniques. *IEEE Trans. Aerosp. Electron. Syst.* **27**, 194–207 (1991).

[18] Margrave, G. F. & Lamoureux, M. P. *Numerical methods of exploration seismology: with algorithms in MATLAB®* (Cambridge University Press, 2019).

[19] Liu, X. *et al.* Non-line-of-sight imaging using phasor-field virtual wave optics. *Nature* **572**, 620–623 (2019).

[20] Liu, X., Bauer, S. & Velten, A. Phasor field diffraction based reconstruction for fast non-line-of-sight imaging systems. *Nat. Commun.* **11**, 1–13 (2020).

[21] O'Toole, M. *et al.* Reconstructing transient images from single-photon sensors. In *Proc. CVPR*, 1539–1547 (2017).

[22] Young, S., Lindell, D. B. & Wetzstein, G. Non-line-of-sight surface reconstruction using the directional light-cone transform. In *Proc. CVPR*, 1407–1416 (2020).

[23] Boyd, S. *et al.* Distributed optimization and statistical learning via the alternating direction method of multipliers. *Found. Trends Mach. Learn.* **3**, 1–122 (2011).

[24] Ikoma, H., Broxton, M., Kudo, T. & Wetzstein, G. A convex 3D deconvolution algorithm for low photon count fluorescence imaging. *Sci. Rep.* **8**, 1–12 (2018).

[25] Parikh, N., Boyd, S. *et al.* Proximal algorithms. *Found. Trends Optim.* **1**, 127–239 (2014).

[26] Landauer, R. & Buttiker, M. Diffusive traversal time: Effective area in magnetically induced interference. *Phys. Rev. B* **36**, 6255–6260 (1987).
