# Supplementary material for: Three-dimensional imaging through scattering media based on confocal diffuse tomography
Source: Nat Commun. 2020 Sep 9;11:4517. doi: 10.1038/s41467-020-18346-3 (PMC7481188; doi:10.1038/s41467-020-18346-3)
Supplement: Supplementary file 3 — Description of Additional Supplementary Files [file 41467_2020_18346_MOESM3_ESM.pdf]

Supplementary file details for

Three-dimensional imaging through scattering media based on confocal diffuse tomography

David B. Lindell, Gordon Wetzstein

Department of Electrical Engineering, Stanford University
350 Jane Stanford Way, Stanford, CA, 94305

Supplementary Movie 1

Description of confocal diffuse tomography. This movie provides a narrated description of the imaging setup and reconstruction algorithm.

Supplementary Movie 2

Visualization of captured results. This movie provides visualizations of captured measurements and reconstructions using confocal diffuse tomography.

Supplementary Data 1

Demonstration code and dataset. This repository contains sample code and data to reproduce the results of Fig. 2 and Fig. 3 of the paper. Instructions and requirements to run the code are described within.
